# Strongly Anharmonic Octahedral Tilting in Two-Dimensional Hybrid Halide Perovskites

**DOI:** 10.1021/acsnano.1c02022

**Published:** 2021-05-18

**Authors:** Matan Menahem, Zhenbang Dai, Sigalit Aharon, Rituraj Sharma, Maor Asher, Yael Diskin-Posner, Roman Korobko, Andrew M. Rappe, Omer Yaffe

**Affiliations:** † Department of Chemical and Biological Physics, Weizmann Institute of Science, Rehovot 76100, Israel; ‡ Department of Chemistry, University of Pennsylvania, Philadelphia, Pennsylvania 19104-6323, United States; § Chemical Research Support, Weizmann Institute of Science, Rehovot 76100, Israel

**Keywords:** 2D perovskites, polarization-orientation, Raman, anharmonicity, phase transition, octahedral tilt, (C_4_H_9_NH_3_)_2_PbI_4_

## Abstract

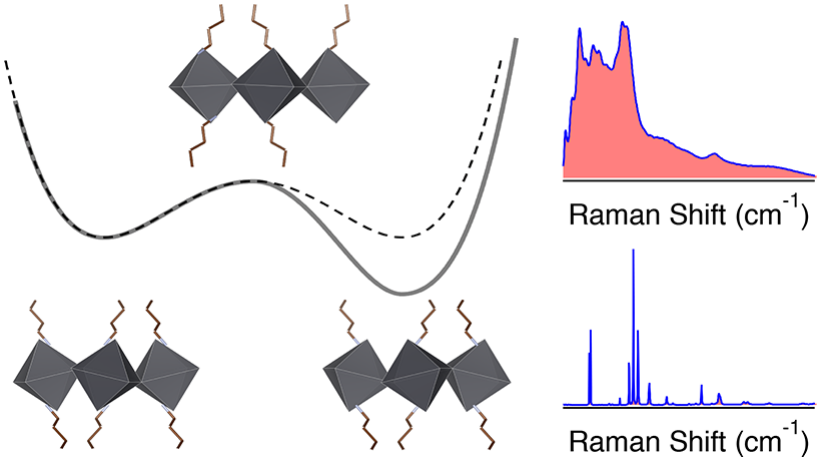

Recent investigations of two-dimensional (2D) hybrid organic–inorganic halide perovskites (HHPs) indicate that their optical and electronic properties are dominated by strong coupling to thermal fluctuations. While the optical properties of 2D-HHPs have been extensively studied, a comprehensive understanding of electron–phonon interactions is limited because little is known about their structural dynamics. This is partially because the unit cells of 2D-HHPs contain many atoms. Therefore, the thermal fluctuations are complex and difficult to elucidate in detail. To overcome this challenge, we use polarization-orientation Raman spectroscopy and *ab initio* calculations to compare the structural dynamics of the prototypical 2D-HHPs [(BA)_2_PbI_4_ and (PhE)_2_PbI_4_] to their three-dimensional (3D) counterpart, MAPbI_3_. Comparison to the simpler, 3D MAPbI_3_ crystal shows clear similarities with the structural dynamics of (BA)_2_PbI_4_ and (PhE)_2_PbI_4_ across a wide temperature range. The analogy between the 3D and 2D crystals allows us to isolate the effect of the organic cation on the structural dynamics of the inorganic scaffold of the 2D-HHPs. Furthermore, using this approach, we uncover the mechanism of the order–disorder phase transition of (BA)_2_PbI_4_ (274 K) and show that it involves relaxation of octahedral tilting coupled to anharmonic thermal fluctuations. These anharmonic fluctuations are important because they induce charge carrier localization and affect the optoelectronic performance of these materials.

Two-dimensional (2D) hybrid (organic–inorganic) halide perovskites (HHPs) have gained renewed attention in recent years for their potential in optoelectronic applications because of their pronounced excitonic properties.^1−4^ 2D-HHPs are layered crystals consisting of corner-sharing metal halide octahedra, separated by bilayers (or monolayers) of organic ammonium cations.^5−7^ Their optical and electronic properties can be readily tuned by modifying the organic side chain.^8−10^ An important example for this tunability is that the type of organic side chain can strongly affect the exciton binding energy *via* dielectric confinement.^11−14^ The most studied 2D-HHPs are butyl-ammonium lead iodide ((BA)_2_PbI_4_) and phenethylammonium lead iodide ((PhE)_2_PbI_4_). These organic cations form high-quality crystals from solution at relatively low temperatures (≈90 °C).^15−17^ The dielectric constant of the saturated, butyl-ammonium molecular layer is significantly smaller than that of the conjugated phenethylammonium layer, leading to larger exciton binding energy.^17−19^ Furthermore, the intermolecular interactions within the organic layers determine the structural phase sequence of the crystals.^5^ Relatively weak interactions between the butyl-ammonium chains result in a structural phase transition at 274 K that is not observed in (PhE)_2_PbI_4_ crystals, even at much higher temperatures.^20−24^

An important aspect that sets 2D-HHPs apart from standard tetrahedrally bonded semiconductors is that they exhibit complex vibrational behavior at terahertz frequencies.^25^ These low-frequency optical phonons are heavily populated at room temperature and can affect the dielectric environment of charge carriers, leading to phonon-assisted scattering, charge carrier screening,^26^ and charge carrier localization.^27,28^ These strong electron–phonon interactions are expected to dominate the electronic and optical properties of the crystals.^29−32^

Despite conclusive indications of large electron–phonon coupling in 2D-HHPs,^27,33−35^ there are only a few reported studies of their structural dynamics. The Raman spectra of (BA)_2_PbI_4_ and (PhE)_2_PbI_4_ were reported, and modes have been assigned to particular ionic displacements.^24,36−40^ First-principles molecular dynamics simulations have shown large rotational and conformational freedom of the organic cations; however, the motions of the optically active inorganic layer were not included in these studies.^41,42^

Contrary to the aforementioned 2D-HHPs, the structural dynamics of their three-dimensional (3D) counterpart, methylammonium lead iodide (MAPbI_3_), was extensively studied.^43−57^ Specifically, we have recently elucidated the microscopic mechanism that leads to strong anharmonicity in MAPbI_3_.^58^

Since (BA)_2_PbI_4_, (PhE)_2_PbI_4_, and MAPbI_3_ all comprise the same metal-halide corner-sharing octahedra, it is likely that the structural dynamics of 2D-HHPs and their 3D counterpart are related. Therefore, we present a study in which we investigate the structural dynamics of (BA)_2_PbI_4_ and (PhE)_2_PbI_4_ by comparing them with MAPbI_3_. We use polarization-orientation (PO) Raman scattering and *ab initio* calculations to extract the vibrational symmetries of 2D-HHPs at low temperature. The results reveal striking similarities between the 2D and 3D crystals. Furthermore, we probe the evolution of the structural dynamics with temperature across the phase transition of (BA)_2_PbI_4_. By comparing MAPbI_3_ and (BA)_2_PbI_4_, we suggest that the phase transition of the latter at 274 K occurs *via* an unlocking of anharmonic octahedral tilting motion, coupled to atomic displacements along the stacking direction. This means that despite the well-defined average structure of (BA)_2_PbI_4_ at room temperature (as extracted from X-ray diffraction),^20^ the octahedra are constantly fluctuating between different tilted configurations. These fluctuations occur on the picosecond time scale and can strongly interact with charge carriers and excitons.

## Results and Discussion

### Crystal Structure

**Figure 1:**
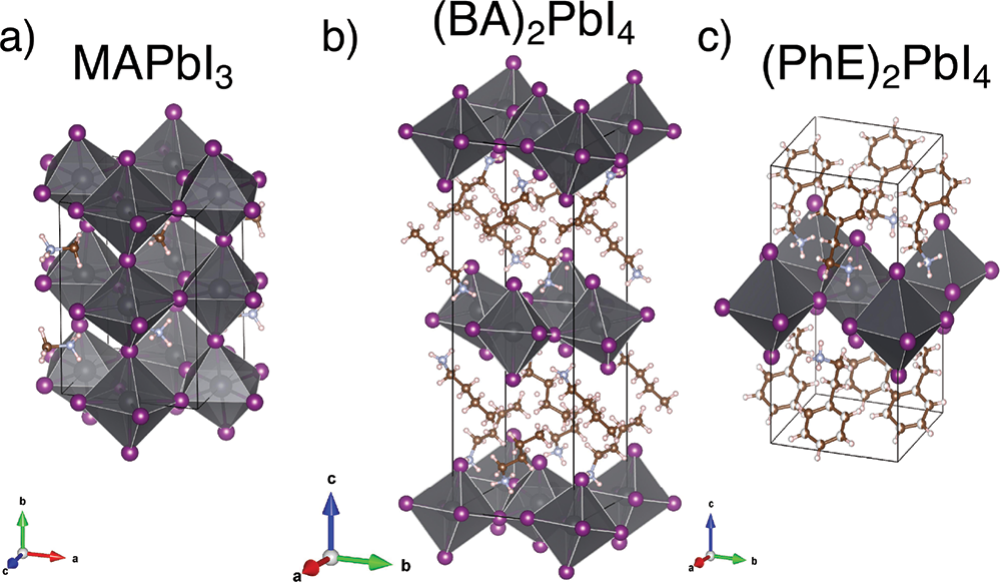
Crystal structures of (a) MAPbI_3_, (b) (BA)_2_PbI_4_, and (c) (PhE)_2_PbI_4_ obtained by single-crystal XRD measurements at 100 K, presented with the longest crystallographic axis as vertical. The structure of MAPbI_3_ was reproduced using a CIF from the Cambridge Structural Database,^65^ CSD 428898, previously reported in ref (55).

Single crystals of (BA)_2_PbI_4_ and (PhE)_2_PbI_4_ were synthesized according to previously reported procedures^16,30,59,60^ (for detailed description, see the Methods section). We start our analysis by comparing the structure of the crystals as refined from single-crystal X-ray diffraction (XRD) at 100 K (*i*.*e*., low-temperature phase of all crystals).^61,62^Figure 1 shows the crystal structure of MAPbI_3_ (left), (BA)_2_PbI_4_ (center), and (PhE)_2_PbI_4_ (right). In the low-temperature phase, MAPbI_3_ and (BA)_2_PbI_4_ are both orthorhombic (*Pnma* and *Pbca* space groups, respectively), while (PhE)_2_PbI_4_ is triclinic (*P*1̅). The alignment and corrugation of the lead iodide octahedra are similar in all crystals, although they are modified by the interactions with the organic ammonium ions between the octahedra (for structural data, see Section S2 in the Supporting Information, SI).^40,54,63,64^ (PhE)_2_PbI_4_ exhibits strong van der Waals interactions between the conjugated rings, forcing the structure to lower symmetry.

**Figure 2:**
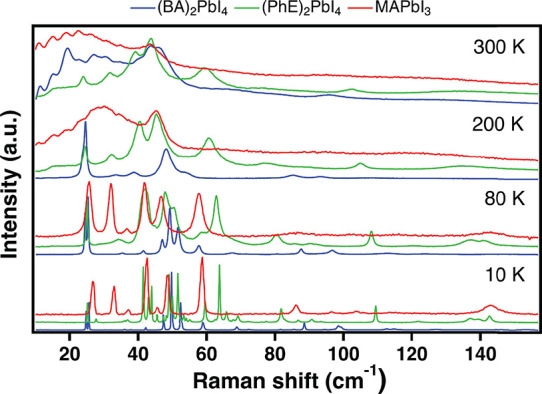
Unpolarized (summing over all excitation polarizations) low-frequency Raman spectra of (BA)_2_PbI_4_ (blue), (PhE)_2_PbI_4_ (green), and MAPbI_3_ (red) at selected temperatures, showing the spectral similarities above 80 K and the mode splitting caused by the organic cations at 10 K.

### Effect of Organic Cation on Structural Dynamics

To uncover the effect of the organic cation on the structural dynamics of the inorganic scaffold, we probe the structural dynamics using Raman scattering. (BA)_2_PbI_4_ and (PhE)_2_PbI_4_ were measured perpendicular to the (001) plane, while MAPbI_3_ was measured perpendicular to the (101) plane (see ref (66)). We limit ourselves to low-frequency vibrations (<150 cm^–1^) because only they carry information about lattice modes.^67,68^

Figure 2 shows the unpolarized Raman spectra (summing over all excitation polarizations) of (BA)_2_PbI_4_ (blue traces), (PhE)_2_PbI_4_ (green traces), and MAPbI_3_ (red traces) at selected temperatures. At 80 K, the observed spectra are similar for all three crystals with respect to both number and frequency of the peaks. The intensity ratios between the peaks differ, due to different scattering cross sections. As a consequence, the peaks at ≈30 cm^–1^ and above 60 cm^–1^ in the spectrum of (BA)_2_PbI_4_ are much lower in intensity (for the list of frequencies, see Table S5 in the SI). As temperature decreases to 10 K, the peaks of MAPbI_3_, (BA)_2_PbI_4_, and (PhE)_2_PbI_4_ blue-shift and sharpen. Furthermore, the peaks of (PhE)_2_PbI_4_ split into multiple sharp peaks. The number of peaks in the 10 K spectrum of (PhE)_2_PbI_4_ exceeds the number predicted from the crystal structure. This indicates that the refined structure at 100 K may not accurately reflect structure at 10 K and cannot account for the observed Raman-active modes (for further discussion, see Section S3 in the SI). The observed mode splitting is related to the stiffness of the crystal, due to strong interactions between the organic molecules of opposite layers.^37^ The organic molecules in MAPbI_3_ are well separated, and their interaction is screened by the iodine and lead atoms. In (BA)_2_PbI_4_, the alkyl-chains interact through weak van der Waals interactions, while in (PhE)_2_PbI_4_ the interactions are stronger due to the conjugated molecules. As the strength of interaction increases, the organic molecule imposes more constraints on the structure^33,69^ which results in lower symmetry.

At 200 K, MAPbI_3_ completes a phase transition to its tetragonal phase (162 K, *I/4mcm*), and the Raman spectrum is significantly broadened (we reported on this phase transition elsewhere),^58^ while the spectra of (BA)_2_PbI_4_ and (PhE)_2_PbI_4_ remain relatively sharp and resolved. Once *T* rises to 300 K, (BA)_2_PbI_4_ has also completed a structural phase transition (274 K, *Pbca*),^20,21^ and its Raman spectrum is strikingly similar to that of MAPbI_3_ at the same temperature. Both exhibit broad features that are centered around 25 cm^–1^ and a pronounced shoulder around 47 cm^–1^. (PhE)_2_PbI_4_ does not exhibit a phase transition, and indeed, its Raman spectrum remains relatively sharp and resolved, even at higher temperatures (for temperature-dependent Raman spectra, see Figure S3 in the SI).

At low temperatures, the organic cation limits the atomic motions of the inorganic network through its intermolecular interactions and causes mode splitting. As temperature increases and intermolecular interactions soften, the structural dynamics of (BA)_2_PbI_4_, (PhE)_2_PbI_4_, and MAPbI_3_ become comparable. In light of the structural and spectral similarities, we turn to investigate further the structural dynamics of (BA)_2_PbI_4_ and MAPbI_3_ using PO Raman scattering, which allows us to assign mode symmetries and investigate the anharmonic behavior of modes.^70^ Henceforth, we focus on (BA)_2_PbI_4_, deferring detailed consideration of (PhE)_2_PbI_4_ to future work. The low-symmetry and complex Raman spectra of (PhE)_2_PbI_4_ at low temperatures hinder the extraction of meaningful conclusions.

### Symmetry Assignment and Anharmonic Behavior *via* PO Raman Scattering

In a previous study, we used PO Raman scattering and *ab initio* molecular dynamics to provide a microscopic picture of the thermal fluctuations of MAPbI_3_ in the tetragonal phase at 300 K.^58^ We showed that the broad Raman spectra are a result of strongly anharmonic octahedral tilting that continuously increases with temperature. The similarities between the spectra of MAPbI_3_ and (BA)_2_PbI_4_ in both the room-temperature and low-temperature phases prompted us to investigate whether the anharmonic octahedral tilting that governs the dynamics and phase transitions in MAPbI_3_ are also present in (BA)_2_PbI_4_. To do so, we conducted temperature-dependent PO Raman measurements on (BA)_2_PbI_4_ and compared them with those of MAPbI_3_. The details of the PO Raman measurements and analysis are discussed in Section S5 in the SI. Briefly, the crystal (oriented perpendicular to the (001) plane) is excited by plane-polarized laser light. The scattered light is then filtered by another polarizer (analyzer) oriented parallel or perpendicular to the incident light polarization. This PO Raman measurement is repeated after each incremental rotation (10°) of the incident light polarization.

**Figure 3:**
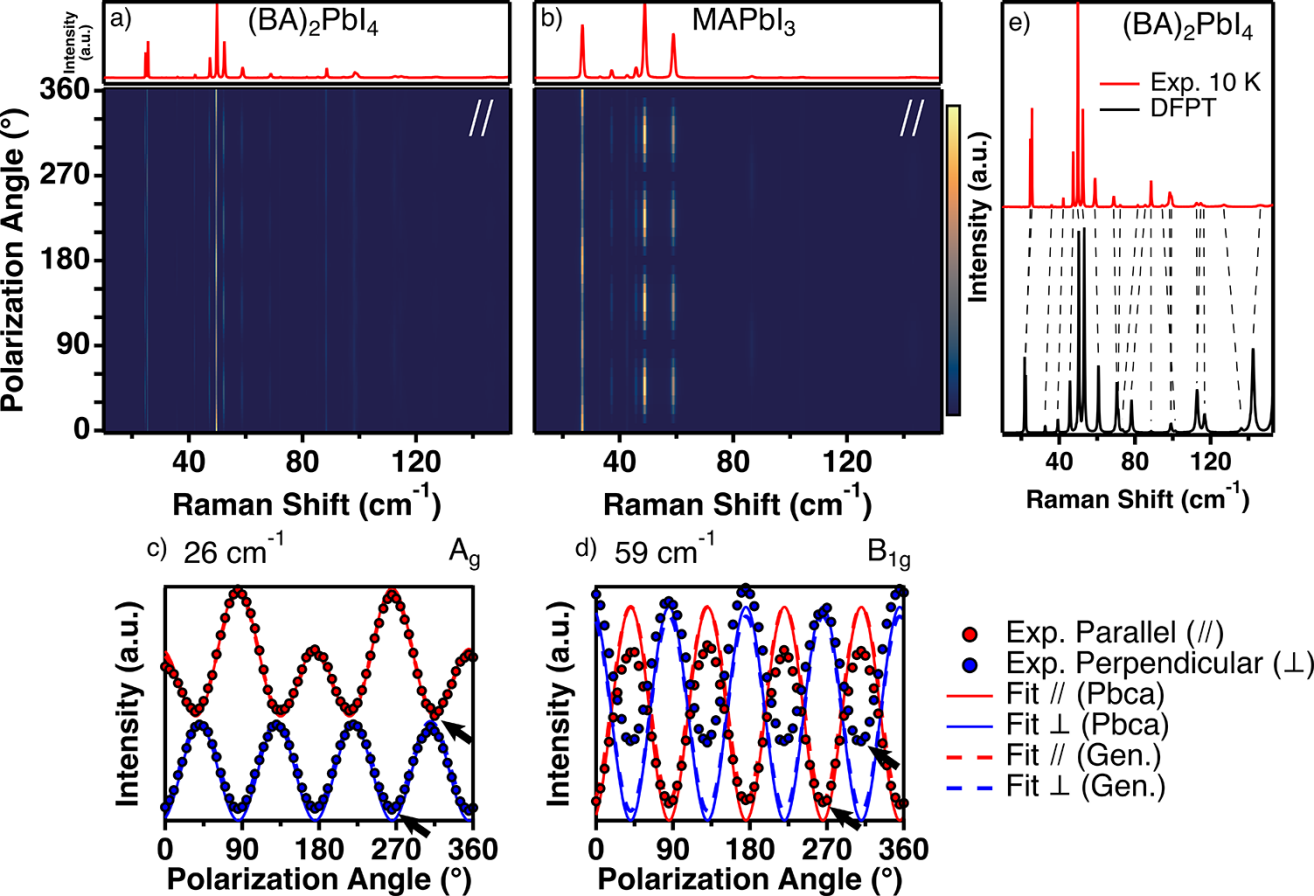
PO Raman spectra of (a) (BA)_2_PbI_4_ and (b) MAPbI_3_ at 10 K in the parallel configuration. Top panel is the corresponding unpolarized Raman spectrum obtained by summing over all polarizations. PO dependence of a typical (c) A_g_ and (d) B_1g_ mode of (BA)_2_PbI_4_, in the parallel (red) and perpendicular (blue) configurations. Experimental data are represented by circles, and the dotted and dashed lines are the best fit to eq 1 using the tensors in eqs 2 and 3, respectively. The arrows mark the nonvanishing intensity at the minima of both A_g_ and B_1g_ modes. (e) Unpolarized Raman spectrum of (BA)_2_PbI_4_ at 10 K compared to that calculated from DFPT, showing excellent agreement.

Since the spectra at 10 K are well resolved, we can use the measured data to assign individual peaks to normal modes and extract the Raman tensor for each mode. Figure 3a,b is contour plots of the PO Raman spectra of (BA)_2_PbI_4_ and MAPbI_3_ at 10 K in the parallel configuration, respectively. (The spectra in Figure 3b were recalculated from data previously reported by us.^66^ For detailed description and plots of the perpendicular configuration, see Figure S5 and Section S7 in the SI.) The top panels of Figure 3a,b presents the corresponding unpolarized spectra (integrated over all angles). The intensity modulations (*i*.*e*., vertical cross sections) observed in the bottom panels of Figure 3a,b represent the intensity angular dependence (PO dependence) of each mode. This PO dependence is dictated by the Raman tensor corresponding to the irreducible representation of each mode.^71−73^ To extract the Raman tensor of each peak, we first fit each individual spectrum to the product of the Bose–Einstein distribution and a multipseudo-Voigt line-shape (for more details, see Section S6 in the SI). We use a pseudo-Voigt line-shape instead of the commonly used damped Lorentz oscillators model^74,75^ because the widths of the peaks at 10 K approach our system resolution, resulting in inhomogeneous broadening which cannot be captured by the Lorentz oscillator line-shape.

We then calculate the integrated intensity of each mode as a function of excitation polarization angle. Finally, we extract the Raman tensor components by fitting the integrated intensity, of both parallel and perpendicular configurations simultaneously, to the modified Placzek equation,^66^ given by
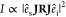
1where *ê*_i_ and *ê*_s_ are the polarization vectors of the incident and scattered light, respectively. **R** is the Raman tensor, and **J** is the Jones matrix,^76,77^ introduced to account for birefringence effects due to anisotropy in the crystal structure.^78−80^ The *ê*_i_ and *ê*_s_ are either parallel or perpendicular to one another, depending on the measurement configuration.

The form of the Raman tensor, as well as the number of expected Raman-active modes, is extracted by performing factor-group analysis^81^ to the relevant space group (orthorhombic, *Pbca*). This analysis is based on the harmonic and rigid-body approximations.^68^ Under the harmonic approximation, we assume no mode coupling, and each normal mode corresponds to a single irreducible representation of the factor group.^82^ Under the rigid-body approximation, we treat the organic molecules as rigid spheres with no internal motions.^68^

Factor-group analysis predicts 48 Raman-active lattice modes for (BA)_2_PbI_4_, 24 of which scatters light polarized in the (001) plane, that is, 12 A_g_ modes and 12 B_1g_ modes, with the corresponding Raman tensors:^83^
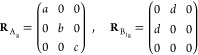
2

Two distinct dependencies are observed for the A_g_ and B_1g_ modes of (BA)_2_PbI_4_ in the parallel configuration, as can be seen in Figure 3c,d. The former (26 cm^–1^) exhibits two peaks with 180° periodicity, while the latter (58 cm^–1^), shifted by 45°, exhibits one peak with 90° periodicity (for mode assignments, see Figure S6 and Table S5 in the SI). A similar distinction was previously observed in the PO Raman of MAPbI_3_.^66^

It is noteworthy that the observed PO of (BA)_2_PbI_4_ is not fully captured by our model. Specifically, contrary to the observation in MAPbI_3_,^66^ the intensity of each peak does not vanish at the minima (Figure 3c,d). This discrepancy indicates that either the rigid-body and/or the harmonic approximations are not fully valid for (BA)_2_PbI_4_. To explore this further, we first explore relaxing the rigid-body approximation. The internal motions of the organic molecules cannot be neglected, and the actual space group of the crystal has lower symmetry than that extracted from XRD measurements. To address the lower symmetry, we repeat our analysis with a completely general Raman tensor:
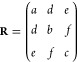
3

We find that the PO dependencies of (BA)_2_PbI_4_ could not be reproduced accurately even with a completely general tensor (dashed line in Figure 3c,d).

We suggest two possible explanations for the observation. The first is that the nonvanishing intensity at the minima indicates that both the harmonic and the rigid body approximations are not valid for (BA)_2_PbI_4_. The second is the formation of small domains in the crystal, which are smaller than our spatial resolution, induced by strain, due to the temperature gradients and different thermal expansion coefficients of the crystal and thermal contacts. Scattering from domains of different orientations would result in the observed nonvanishing intensity. Measurements at different spots on the crystal show a higher intensity ratio between the minima and maxima, as we move closer to the thermal contacts. This supports the claim of microdomains formation.

### Real-Space Assignments and Mode Correlation

After assigning a symmetry to each peak in the spectra, we set about obtaining real-space assignments of the modes of (BA)_2_PbI_4_. To that end, we used *ab initio* density functional perturbation theory (DFPT) analysis following a previously published procedure.^66^Figure 3e shows the unpolarized spectrum of (BA)_2_PbI_4_ obtained from DFPT calculations, compared to the experimental spectrum at 10 K. We find excellent agreement between the experimental and calculated spectra, which allows us to assign the modes of (BA)_2_PbI_4_ according to frequency and symmetry and extract the real-space assignment of each mode. Evidently, all the calculated Raman-active normal modes of (BA)_2_PbI_4_ contain significant contributions of libration motion of the organic cation.

Combining the obtained frequencies, symmetries, and real-space assignments, we can correlate between the modes of (BA)_2_PbI_4_ and MAPbI_3_. The detailed comparison and real-space assignments are given in the Table S6 in the SI. Despite the large contribution of organic cations, the modes’ symmetries and real-space assignments are strikingly similar, highlighting the intimate relationship between the structural dynamics of 2D and 3D lead iodide HHPs.

### Structural Dynamics in the High-Temperature Phase

**Figure 4:**
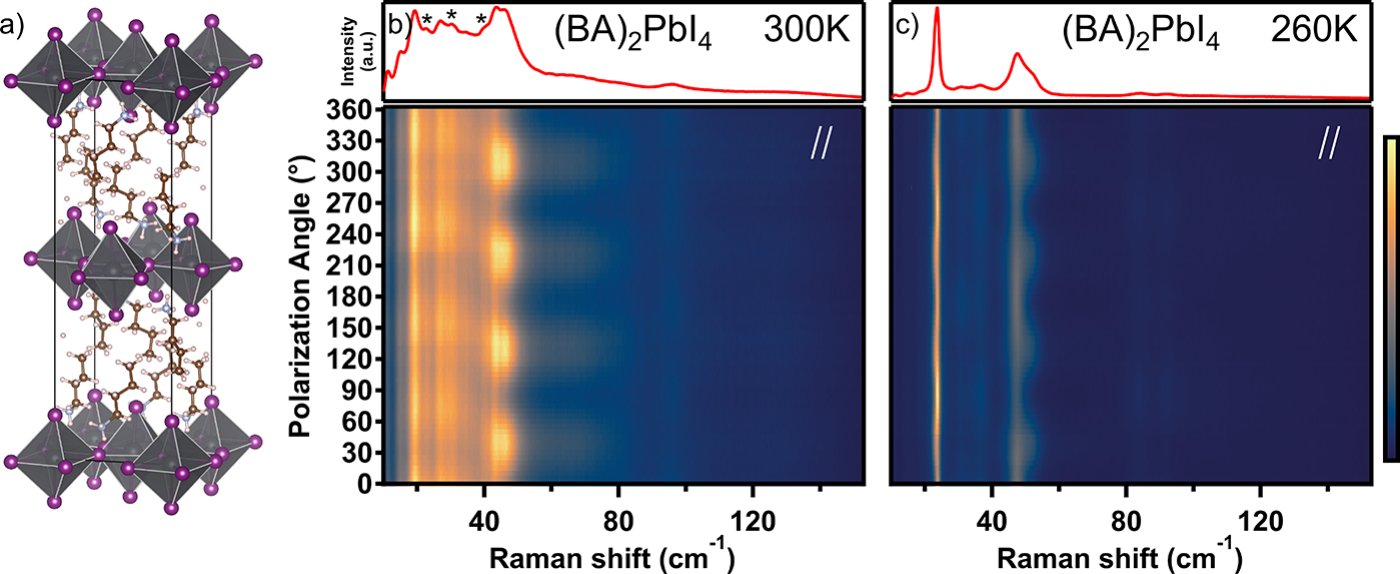
(a) Crystal structure of (BA)_2_PbI_4_ obtained by single-crystal XRD measurements at room temperature. PO Raman spectra of (BA)_2_PbI_4_ (b) at 300 K and (c) 260 K in the parallel configuration. Top panel is the corresponding unpolarized spectrum obtained by summing over all polarizations. Modes marked with * are A_g_ modes which could not be uniquely associated with modes of the low-temperature phase.

We have established that the structural dynamics of the low-temperature phases of (BA)_2_PbI_4_ and MAPbI_3_ are closely related. We now turn to focus on the structural dynamics of (BA)_2_PbI_4_ at room temperature. Figure 4a presents the crystal structure refined from XRD at room temperature. Figure 4b,c presents the PO Raman spectra, in the parallel configuration (perpendicular configurations presented in Figure S7 in the SI), just above and below the phase transition, at 300 and 260 K. The top panel shows the unpolarized spectra. It should be noted that there is a known hysteresis in the phase transition temperature of (BA)_2_PbI_4_.^20^ Nevertheless, the same XRD crystal structure and the same trend in the Raman spectra were observed (around the phase transition temperature) when heating or cooling the crystal across the phase transition.

The crystal structures above and below the phase transition are both orthorhombic with the same space group, *Pbca*.^20^ In the high-temperature phase, we observe some reduction in octahedral corrugation angles. The angle between the butylammonium cations and the Pb–I plane approaches 90°, and the interlayer spacing increases (for structural data, see Table S4 in the SI). These slight differences have been shown to have a significant effect on the electronic properties of (BA)_2_PbI_4_.^17,84−86^

As discussed for the low-temperature phase, factor-group analysis predicts 24 Raman-active modes for the high temperature phase, 12 A_g_ and 12 B_1g_. However, unlike for the low-temperature phase, the number of observed peaks in Figure 4b,c is considerably smaller, and the shape of the Raman spectrum in Figure 4b is significantly different. The relatively well-defined modes of the lower-temperature phase are replaced by broad overlapping features which are harder to distinguish. Therefore, spectral deconvolution into damped Lorentz oscillators becomes very challenging.

To overcome this challenge, we make use of the similarity to the spectra below the phase transition temperature, as in Figure 4c. We begin by assigning all the modes below the phase transition. To that end, we follow the temperature evolution and symmetry of the modes from 10 K (for more PO Raman spectra, see Figure S7 in the SI) and assigned all the observed modes at 260 K. Then, we compare the spectral features and their PO dependencies to those at 300 K (Figure 4a,b and Figure S7 in the SI) and assign the modes above the phase transition. The most pronounced change in the spectra, due to the phase transition, is the broadening of low-frequency A_g_ modes with a significant octahedral tilting component around the crystallographic *b* axis.

Interestingly, three weak, low-frequency A_g_ modes, observed at 300 K, could not be directly assigned to modes of the low-temperature phase. These modes exhibit the same PO dependence as the A_g_ modes adjacent to them (see Figure S8 in the SI). The higher number of A_g_ modes in this low-frequency range at the high-temperature phase, their PO dependencies, and the lack of change in space-group during the phase transition pose the question of the origin of these modes. The Raman active modes in (BA)_2_PbI_4_ are nondegenerate, therefore mode splitting due to degeneracy removal cannot occur. Scattering from different domains would not account for the frequency shift of the modes, while stress related scattering would be more prominent in the low-temperature phase. Nevertheless, the similar PO dependencies and frequencies suggest that these modes and their adjacents originate from a very similar atomic motion. We propose that these modes are actually the same and are a signature of the phase transition mechanism, which will be discussed later.

The successful assignment of the modes and their similar symmetries suggests that the low- and high-temperature phases exhibit similar atomic displacements, with larger amplitudes and shorter lifetimes above the phase transition temperature (for mode assignment across the temperature range, see Table S5 in the SI). This is an important finding because XRD measurements show that the average crystal structure has changed (Figures 1b and 4a). The Raman measurements provide additional insight, showing that the main change in the phase transition is a dynamic change where the thermal fluctuations become strongly damped (*i.e*. broad peaks), soft (*i.e*. peaks shift to lower frequencies), and thus, strongly anharmonic. Therefore, we now turn to elucidating the mechanism of the phase transition.

### Mechanism of Phase Transition in (BA)_2_PbI_4_

**Figure 5:**
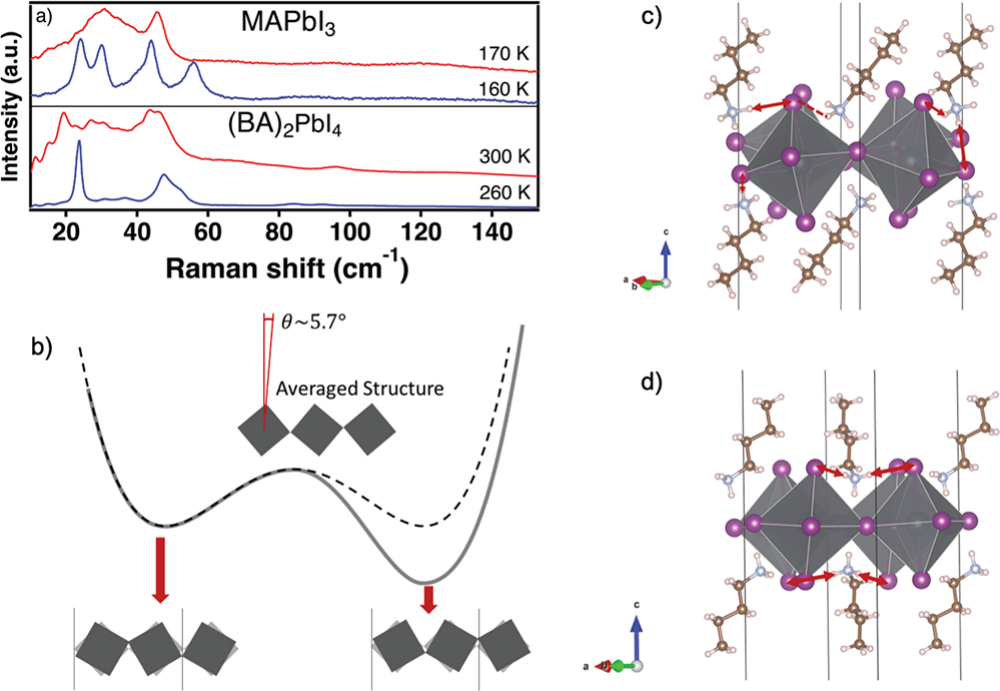
(a) Unpolarized Raman spectra of MAPbI_3_ and (BA)_2_PbI_4_ below (blue traces) and above (red traces) the phase transition. The measurements’ temperatures are indicated above the traces, and the spectra are offset for clarity. (b) Illustration of asymmetric double-well potential of (BA)_2_PbI_4_ due to the hydrogen bonding. The dashed-line is the conventional symmetric double well. Above 274 K, the relaxational octahedral rotation is activated, and both wells are populated, but the average structure has a finite tilt angle. (c) Illustration of the hydrogen bonds in the low-temperature phase of (BA)_2_PbI_4_. The lower kinetic energy combined with stronger hydrogen bonds locks the structure with a large octahedral tilting angle. (d) Illustration of the hydrogen bonds in high-temperature (BA)_2_PbI_4_. The relaxational octahedral rotation is activated at this phase, but the hydrogen bonding to the BA molecules makes the relaxational rotation asymmetric.

We recently reported the mechanism of the orthorhombic-to-tetragonal phase transition in MAPbI_3_.^58^ Our main conclusions were that two types of motions are causing the phase transition: the orientational unlocking of the [CH_3_NH_3_]^+^ ions and large amplitude octahedral tilting that continuously increases with temperature. It was previously reported that the phase transition of (BA)_2_PbI_4_ is also related to an order–disorder transition of the organic molecules.^5,17,61,87^ However, the role of the octahedral tilting was not explored. In Figure 5a, we compare the abrupt change in the Raman spectra of (BA)_2_PbI_4_ and MAPbI_3_ due to the phase transition. The blue and red traces are the unpolarized Raman spectra below and above the phase transition, respectively. The change in the Raman spectra at the phase transition in (BA)_2_PbI_4_ and MAPbI_3_ is similar. This similarity leads us to suggest that, also in (BA)_2_PbI_4_, the mechanism for the phase transition involves unlocking of large amplitude, anharmonic octahedral tilting.

This means that similar to MAPbI_3_ and other halide perovskites,^88−91^ the high-temperature phase of (BA)_2_PbI_4_ has the relaxational octahedral tilting activated, and the structure is in a steady state in which it visits different octahedral tilting orientations. Figure 5b presents the order–disorder phase transition mechanism as the population of a double-well potential, where each represents a different tilting orientation.

According to the order–disorder phase transition mechanism (dashed line in Figure 5b), we would expect the time-averaged structure to be untilted.^92^ However, the finite tilt angle observed in Figure 4a suggests an additional displacive component to the phase transition. We interpret the displacive component as an asymmetry in the double-well,^93^ as presented in Figure 5b. This asymmetry is caused by the hydrogen bonding between [NH_3_]^+^ group and the iodides. Unlike the isolated MA molecules in MAPbI_3_ where a “free” rotation is allowed, the BA molecules have a preferred configuration due to their intermolecular interactions. This orientation results in an anisotropic bonding network with the iodides, even at the high temperature phase. Figure 5c,d illustrates the hydrogen-bonding network in the low- and high-temperature phases, respectively. Such anisotropic bonding imposes a bias on the double-well. Therefore, the low-temperature phase is locked in a large tilting angle due to the lower kinetic energy, while the high-temperature phase exhibits a smaller but finite tilting angle as well. Another indication for the biased double-well potential is the extra A_g_ modes, observed in the high-temperature phase, unassociated uniquely to modes of the low-temperature phase (marked peaks in Figure 4b). These modes exhibit the same PO dependence as their adjacent A_g_ modes, suggesting they originate from the same motion in a different low-temperature phase, that is, a different well. The energy-shift between the adjacent A_g_ modes is a result of the bias between the wells in the potential.

On the other hand, like in MAPbI_3_, the relaxational octahedral rotation is coupled to the vibrational modes at the Γ point, effectively damping them and leading to the drastic broadening of the peaks observed in the Raman spectra during phase transition.^58^ As mentioned earlier, according to our mode assignment, these are mainly low-frequncy A_g_ modes with a significant octahedral tilting component around the crystallographic *b* axis. This type of disorder manifests itself in XRD through thermal factors and would not be apparent in the refined structure (Figure 4a). This is due to the elastic scattering nature of XRD, which gives a higher significance to the average structure over other configurations.

The presence of large-amplitude anharmonic octahedral tilting is also important for the optoelectronic properties of the crystals. These large lattice motions can stabilize localized electronic excited states and are likely to reduce the charge carrier mobility of the materials.^7,94−97^ These fluctuations also contribute to the screening of point defects,^9,98^ and it is of interest to examine in the future whether the optically/electronically active defect density decreases in the high-temperature phase of (BA)_2_PbI_4_ compared to the low-temperature phase.

## Conclusions

We used temperature-dependent PO Raman scattering and DFPT calculations to show the intimate relationship between the structural dynamics of the 2D halide perovskites (BA)_2_PbI_4_ and (PhE)_2_PbI_4_ and their 3D counterpart MAPbI_3_. They exhibit similar Raman mode frequencies and atomic motions related to the inorganic framework, which are split by the intermolecular interactions of the organic cation. These relationships can be extended to other 2D-HHPs and their 3D counterparts and assist in the design of complex optoelectronic devices.

Additionally, we revealed the mechanism for the phase transition of (BA)_2_PbI_4_, showing that it involves the relaxational motion of anharmonic octahedral tilting coupled to octahedral tilting around the *b* axis. Our interpretation suggests a mixed displacive and order–disorder phase transition, that is, a biased double-well potential. This is a clear indication of temperature-activated anharmonicity in 2D-HHPs which can directly confer temperature dependence on the optoelectronic properties and electron–phonon interactions observed in these materials.

## Methods

### Synthesis

#### Materials and Precursors

PbO (99%), phosphinic acid (50%w in H_2_O), 2-phenethylamine (99%), and *n*-butylamine (99.5%) were purchased from Merck, hydriodic acid (stabilizer free, 57%w in H_2_O) was purchased from Holland Moran, and diethyl ether (BHT stabilized) was purchased from Bio-Lab Chemicals. The reagents were used without further treatment.

Pb precursor was prepared by dissolving PbO in a mixture of hydriodic acid and phosphinic acid, on a hot-plate set to 110 °C, under magnetic stirring, to a clear yellow solution.

Organic precursor was prepared in a separate beaker in an ice bath, by reacting the amine solution (1:1 molar ratio to Pb) with high excess of hydriodic acid, while stirring.

#### (PhE)_2_PbI_4_ Single Crystals

Single crystals were synthesized by adding the phenethylamine precursor solution dropwise to the Pb precursor solution. Orange powder was immediately observed in the vial. The magnetic stirring was continued until the solution was clear yellow again. Hydriodic acid was added dropwise (≈1 mL) to speed up the dissolution. The stirring was stopped, and the beaker was capped, placed in a silicon-oil-bath system, and cooled to room temperature at a rate of 2 K/h. The orange plate-like crystals were further quenched in an ice bath, vacuum filtered, washed with diethyl ether, and dried at 90 °C in a vacuum oven for 24 h prior to use.

#### (BA)_2_PbI_4_ Single Crystals

Single crystals were synthesized by adding the butylamine precursor solution dropwise to the Pb precursor solution. Orange powder was observed in the vial by the end of the addition. The vial was then capped, and the hot-plate temperature was increased to 140 °C. The magnetic stirring was continued until the solution was clear yellow. After additional ≈20 min, the magnetic stirrer was removed, and the beaker was placed capped in a silicon-oil-bath system preheated to 105 °C and cooled to room temperature, at a rate of 1 K/h. The oil bath was covered with aluminum foil to maintain uniform temperature. The orange plate-like crystals of (BA)_2_PbI_4_ were collected from the bottom of the beaker by evacuating the supernatant and dried gently and thoroughly with a blotting paper. The crystals were then further evacuated overnight in a glovebox’s antichamber and stored in an N_2_-filled glovebox or a desiccator.

Exact molar ratios between the materials can be found in Table S1 in the SI.

### Single Crystal X-ray Diffraction

Description of the X-ray diffraction measurements as well detailed results and structures can be found in the SI.

### Raman Scattering

Raman scattering measurements were conducted in a home-built backscattering system using a 1.58 eV CW pumped-diode laser (Toptica Inc., USA) and a 1.16 eV CW Nd:YAG laser (Coherent Inc., USA) with 1 mW excitation power (for detailed description of the system and measurement, see Section S5 in the SI).

(BA)_2_PbI_4_ and (PhE)_2_PbI_4_ crystals were mechanically exfoliated^99^ prior to use.

### DFPT Calculation

The nonresonant Raman tensor is evaluated by DFPT^100^ according to the method reported in ref.^66^ The structure is a √2 × √2 × 2 supercell, as resolved by experiments. The lattice parameters are taken as (8.28 Å, 8.94 Å, 25.44 Å) with a 4 × 4 × 2 *k*-mesh. All atomic positions are relaxed before any further calculation. The norm-conserving pseudopotential^101^ is used with the generalized gradient approximation^102^ in combination with the Grimme-d3 van der Waals correction.^103^ The convergence parameters are the same as those in ref (66). All the first-principles calculations are carried out with the Quantum Espresso program.

## Supporting Information Available

Further synthesis description, XRD measurements information, temperature-dependent and PO Raman spectra, computational details, and modes correlation (PDF)
The crystal structure of (BA)_2_PbI_4_ at the low temperature phase (100 K, *Pbca*), from single crystal XRD. Deposited to the Cambridge Crystallographic Data Center, depository number CSD 2018893 (CIF)
The crystal structure of (BA)_2_PbI_4_ the low temperature phase, just below the phase transition (270 K, *Pbca*), from single crystal XRD. Deposited to the Cambridge Crystallographic Data Center, depository number CSD 2018894 (CIF)
The crystal structure of (BA)_2_PbI_4_ at the high temperature phase (293 K, *Pbca*), from single crystal XRD. Deposited to the Cambridge Crystallographic Data Center, depository number CSD 2018895 (CIF)
The crystal structure of (PhE)_2_PbI_4_ at 100 K (*P*1̅), from single crystal XRD. Deposited to the Cambridge Crystallographic Data Center, depository number CSD 2018896 (CIF)
The crystal structure of (PhE)_2_PbI_4_ at 297 K (*P*1̅), from single crystal XRD. Deposited to the Cambridge Crystallographic Data Center, depository number CSD 2018897 (CIF)
The crystal structure of (PhE)_2_PbI_4_ at 400 K (*P*1̅), from single crystal XRD. Deposited to the Cambridge Crystallographic Data Center, depository number CSD 2018898 (CIF)

## Supplementary Material

nn1c02022_si_001.pdf

nn1c02022_si_002.cif

nn1c02022_si_003.cif

nn1c02022_si_004.cif

nn1c02022_si_005.cif

nn1c02022_si_006.cif

nn1c02022_si_007.cif
