# Supplementary material for: Strongly Anharmonic Octahedral Tilting in Two-Dimensional Hybrid Halide Perovskites
Source: ACS Nano. 2021 May 18;15(6):10153–62. doi: 10.1021/acsnano.1c02022 (PMC8223479; doi:10.1021/acsnano.1c02022)
Supplement: Supplementary file 1 — nn1c02022_si_001.pdf [file nn1c02022_si_001.pdf]

Supporting Information

Strongly Anharmonic Octahedral Tilting in Two-Dimensional Hybrid Halide Perovskites

Matan Menahem,[1] Zhenbang Dai,[2] Sigalit Aharon,[1] Rituraj Sharma,[1] Maor Asher,[1] Yael Diskin-Posner,[3] Roman Korobko,[1] Andrew M. Rappe,[2] and Omer Yaffe[1]

[1]*Department of Chemical and Biological Physics, Weizmann Institute of Science, Rehovot 76100, Israel*

[2]*Department of Chemistry, University of Pennsylvania, Philadelphia, Pennsylvania 19104–6323, USA*

[3]*Chemical Research Support, Weizmann Institute of Science, Rehovot 76100, Israel*

S1. Synthesis

As mentioned in the main text, single crystals of $(BA)_2PbI_4$ were synthesized according to a previously reported procedure.[1,2] The procedure was modified for $(PhE)_2PbI_4$ according to similar reported procedures.[3,4]

Table S1 presents an example of amounts used in the synthesis of $(BA)_2PbI_4$ and $(PhE)_2PbI_4$, as described in the Methods section of the main text.

Table S1. Materials' amounts used in the synthesis

Materials		$(BA)_2PbI_4$	$(PhE)_2PbI_4$
Beaker 1	PbO [mmol]	5.045	1.34
	H_3PO_2 [μL]	850	230
	HI [mL]	5.0	1.3
Beaker 2	Organic amine [mmol]	5.045	1.34
	HI [mL]	3.0	11.0

S2. X-ray diffraction

The crystal structure and symmetry of the synthesized $(BA)_2PbI_4$ and $(PhE)_2PbI_4$ crystals were resolved using single-crystal X-ray diffraction (XRD). The refined structures can be found in the CIFs attached to the SI.

The synthesized crystals were immersed in minimal amounts of paratone oil ($(BA)_2PbI_4$) or epoxy ($(PhE)_2PbI_4$) and mounted onto a Mitogen loop, at room temperature.

Data were collected at room temperature and then the crystals were cooled down to 100 K at a rate of $1K/min$. A complete data-set was collected again at 100 K. The crystals were warmed at a rate of $1K/min$ and a complete data-set was collected again at various temperatures. Crystallographic data at the different temperatures is summarized in Tables S2 and S3.

Data were collected on Rigaku Synergy-S dual source diffractometer equipped with Dectris Pilatus3 R CdTe 300 K detector and microfocus, with MoKα (λ=0.71073 Å) with ω scans. Data were integrated with CrysAlis^{PRO} and Gaussian absorption was applied taking into account the crystal size. Structures were solved using SHELXT and further refined with SHELXL by full matrix least-squares refinement based on F^2. All atoms are aniosotropic and hydrogens were calculated in a riding mode.

Figures S1 and S2 present the crystal structures of $(BA)_2PbI_4$ and $(PhE)_2PbI_4$ at the

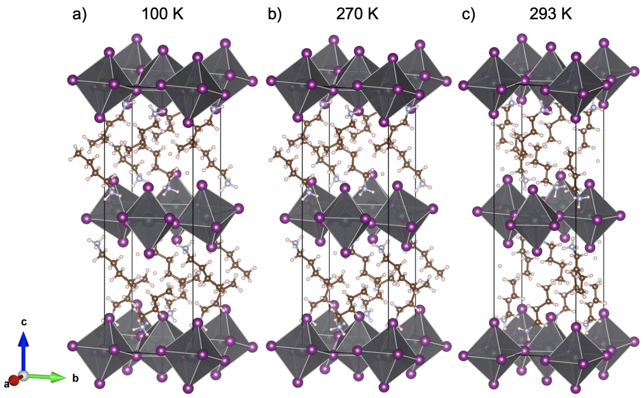

Figure S1. Crystal structure of $(BA)_2PbI_4$ at (a) 100 K, (b) 270 K and (c) 293 K as obtained by single-crystal XRD measurements

various temperatures, respectively. Both $(BA)_2PbI_4$ and $(PhE)_2PbI_4$ maintain the same space-group across the measured temperature range. The difference in the structure of $(BA)_2PbI_4$ at 270 K and room temperature is mainly the alignment of the butyl-ammonium cations and octahedra. This points to the phase transition observed at 274 K.[5]

In order to better understand the structural differences, as well as the octahedral corrugation and alignment, we calculated some distances and angles in the unit-cells of $(BA)_2PbI_4$ and $(PhE)_2PbI_4$. Table S4 presents the calculated bond-lengths and angles between 100 K and room temperature. In parentheses are the standard deviations, as presented by VESTA or calculated by us.

We calculated the number of atoms in the primitive unit-cell by relating atoms in the unit-cell which are related by symmetry and counting the number of repeating chemical formulas. In this way, we find that the unit-cell of $(BA)_2PbI_4$ contain 4 repeating chemical formulas, while the chemical formula of $(PhE)_2PbI_4$ repeats twice in the unit-cell. The

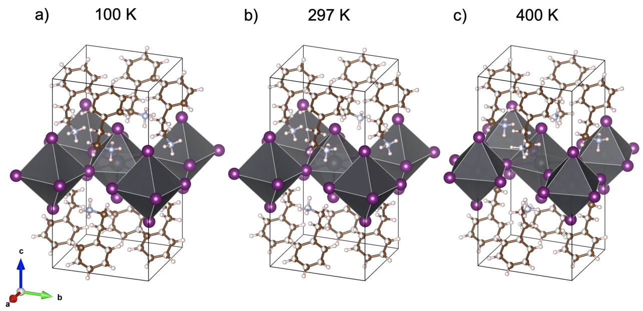

Figure S2. Crystal structure of $(PhE)_2PbI_4$ at (a) 100 K, (b) 297 K and (c) 400 K as obtained by single-crystal XRD measurements

Table S2. Single crystal XRD results of $(BA)_2PbI_4$.

Temperature (K)	100(2)	270(2)	293(2)
CCDC	2018893	2018894	2018895
Chemical Formula		$I_4Pb + 2C_4H_{12}N$	
Formula weight		863.08	
Crystal system		Orthorhombic	
Space group		$Pbca$	
Crystal form and color		Cube, Orange	
Crystal size(mm)	0.090x0.074 x0.044	0.094x0.084 x0.062	0.115x0.085 x0.059
a (Å)	8.4223 (2)	8.5057 (3)	8.8763 (3)
b (Å)	8.9958 (2)	9.0045 (4)	8.6936 (3)
c (Å)	26.0751 (6)	26.6249 (12)	27.6162 (13)
α (°),β (°),γ (°)	90,90,90	90,90,90	90,90,90
Volume (Å^3)	1975.59 (8)	2039.19 (15)	2131.06 (14)
Diffractometer		Rigaku Synergy-S	
Wavelength (Å)		0.71073	
Z		4	
D (Mg m^{-3})	2.902	2.81	2.690
μ (mm^{-1})	14.774	14.313	13.696
Absorption Correction		Gaussian	
T_{min}	0.350	0.900	0.378
T_{max}	0.594	1.000	0.532
Reflections Collected (unique)	14372 (2029)	14997 (2085)	16631 (2184)
R_{int}	0.530	0.0618	0.0314
Completeness (%)	100	100	100
Data / restraints / parameters	2029 / 0 / 72	2085 / 0 / 72	2184 / 1 / 73
Goodness-of-fit on F^2	1.111	1.045	1.071
Final R [I > 2σ(I)], $wR(F^2)$	0.0339, 0.0883	0.0413, 0.1011	0.0256, 0.0586
R (all data), $wR(F^2)$	0.0382, 0.0911	0.0527, 0.1065	0.0352, 0.0616

number of atoms in the unit-cell under the rigid body approximation, *i.e.* considering each organic cation as a rigid sphere, is also presented in Table S4.

In-plane distortion was calculated as the Pb-I-Pb angle, where the location of the bridging iodine (I_B) is projected onto the plane of the Pb atoms (c = 0, 0.5 or 1). Out of plane distortion is the Pb-Pb-I angle between Pb atoms at $c = 0$ and $c = 1$ planes and a terminal iodine (I_T) bound to one of them.[6] We also calculated the width of the organic and in-organic layers, the Pb-I_B and Pb-I_T bond lengths, the average distance between the N atoms and the three nearest I atoms and the penetration of the NH_3 group into the Pb-I layer, *i.e.* the distance between the N atom and the plane of I_T atoms. The penetration

Table S3. Single crystal XRD results of $(PhE)_2PbI_4$.

Temperature (K)	100 (2)	297 (2)	400 (2)
CCDC	2018896	2018897	2018898
Chemical Formula		$I_4Pb + 2C_8H_{12}N$	
Formula weight		959.16	
Crystal system		Triclinic	
Space group		$P\bar{1}$	
Crystal form and color		Plate, Orange	
Crystal size (mm)	0.262x0.193 x0.055	0.290x0.189 x0.051	0.264x0.189 x0.050
a (Å)	8.6735 (2)	8.8764 (2)	8.7708 (2)
b (Å)	8.6816 (1)	8.7374 (2)	8.7724 (2)
c (Å)	16.3969 (4)	16.6556 (4)	16.8772 (3)
α (°)	95.204 (2)	94.425 (2)	95.664 (2)
β (°)	100.503 (2)	99.789 (2)	99.169 (2)
γ (°)	90.562 (1)	90.341 (2)	90.273 (2)
Volume (Å^3)	1210.02 (4)	1247.41 (5)	1275.42 (5)
Diffractometer		Rigaku Synergy-S	
Wavelength (Å)		0.71073	
Z		2	
D (Mg m^{-3})	2.633	2.554	2.498
μ (mm^{-1})	6.515	6.320	6.181
Absorption Correction		Gaussian	
T_{min}	0.361	0.073	0.591
T_{max}	1.000	1.000	1.000
Reflections Collected (unique)	19927 (4956)	20534 (5103)	20940 (5205)
R_{int}	0.0314	0.0492	0.0448
Completeness (%)	100	99.9	99.9
Data / restraints / parameters	4956 / 0 / 265	5103 / 187 / 264	5205 / 227 / 311
Goodness-of-fit on F^2	1.076	1.026	1.101
Final R [I > 2σ(I)], $wR(F^2)$	0.0187, 0.0410	0.0284, 0.0702	0.0326, 0.0824
R (all data), $wR(F^2)$	0.0222, 0.0424	0.0368, 0.0752	0.0458, 0.0918

of the NH_3 group is related to the strength of hydrogen bonding to the I atoms, and was found to strongly affect the in-plane distortion in 2D hybrid halide perovskites.[6]

Table S4. Geometrical parameters in the unit-cell of $(BA)_2PbI_4$ and $(PhE)_2PbI_4$ between 100 K and room temperature. In parentheses are the standard deviations as calculated or printed by VESTA. The calculations are explained in the text.

Material	$(BA)_2PbI_4$			$(PhE)_2PbI_4$	
Temperature (K)	100	270	293	100	297
#Atoms / Rigid-body approx.		156 / 28		94 / 14	
In-plane ∡Pb-I-Pb (°)	148.469 (17)	149.86 (3)	155.018 (15)	152.2 (7)	153.3 (4)
In-plane distortion (°)	157.193 (16)	158.43 (3)	155.361 (15)	152.2 (7)	153.3 (4)
Out-plane distortion (°)	12.859 (9)	12.595 (14)	5.743 (10)	12 (2)	12 (2)
Organic layer (Å)	19.867 (3)	20.404 (3)	21.218 (3)	9.992 (3)	10.2409 (6)
Inorganic layer (Å)	6.208 (3)	6.221 (3)	6.398 (3)	6.404 (2)	6.4147 (1)
Pb-I_B (Å)	3.201 (3)	3.207 (1)	3.181 (3)	3.16 (1)	3.175 (7)
Pb-I_T (Å)	3.1841 (6)	3.1873 (6)	3.2052 (6)	3.202 ()	3.2073 (1)
N-I_T (Å)	3.62 (2)	3.65 (3)	3.63 (2)	3.65 (5)	3.67 (7)
N Penetration (Å)	0.57235 (1)	0.56258 (1)	0.59596 (1)	0.59570 (2)	0.59061 (2)

S3. Temperature-Dependent Raman Scattering

Temperature-dependent Raman measurements were conducted in a home-built back-scattering system, described in details in section S5 below. Crystals were measured using CW laser (1.58 eV for $(BA)_2PbI_4$ and $(PhE)_2PbI_4$, 1.16 eV for $MAPbI_3$) with 1 mW excitation power. $MAPbI_3$ was measured in the absence of polarizers in the system, while $(BA)_2PbI_4$ and $(PhE)_2PbI_4$ were measured in the polarized Raman configuration, at three different excitation angles, in the parallel configuration, where the intensity of the A_g and B_{1g} modes peak (verified by full PO measurement, see section S5). The spectra at each temperature were summed and normalized to obtain the unpolarized spectrum. This procedure results in an equivalent spectrum to the unpolarized spectrum described in section S5.

Figure S3 presents the temperature-dependent unpolarized Raman spectra of $(BA)_2PbI_4$ $(PhE)_2PbI_4$ and $MAPbI_3$. The abrupt change in the Raman spectra of $(BA)_2PbI_4$ at 270 K is indicative of a phase transition. The transition temperature differ from the reported temperature[5] (274 K) due to slight laser heating, despite the fact the laser energy is below the optical band-gap[7–9] (≈2.55 eV). As temperature decreases, clear blue-shifting and sharpening, due to thermal contraction, is observed. The modes of $(PhE)_2PbI_4$ significantly split when temperature approaches 10 K (*e.g.* the mode at 42 cm^{-1} at 80 K splits to

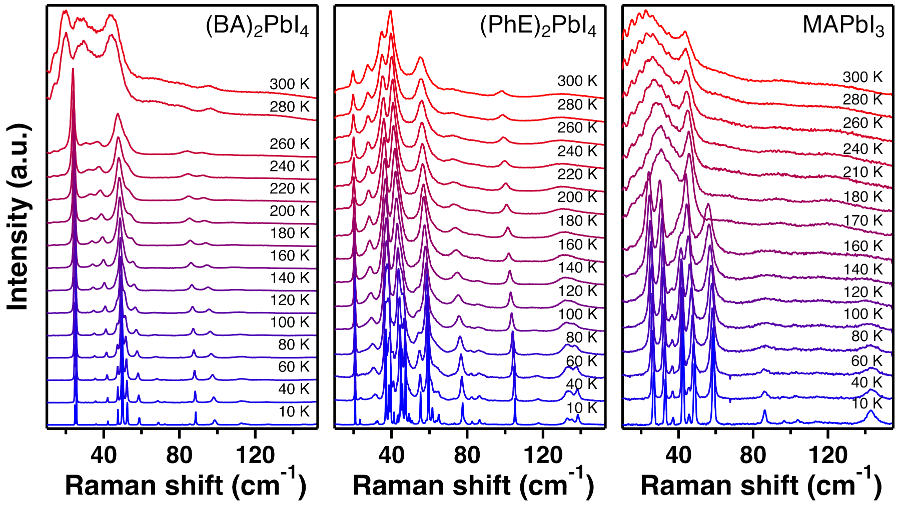

Figure S3. Temperature-dependent unpolarized low-frequency Raman spectra of $(BA)_2PbI_4$ (left), $(PhE)_2PbI_4$ (middle) and $MAPbI_3$ (right) showing the mode splitting when approaching 10 K. The spectra are normalized and offset for clarity.

4 modes between 40.5-44.5 cm^{-1} at 10 K), probably due to the low symmetry and crystal stiffness.

Under the rigid body approximation, factor group analysis predicts 24 Raman active modes for $(BA)_2PbI_4$ and $(PhE)_2PbI_4$, which scatters light polarized in the (001) plane. The larger number of modes observed in the Raman spectrum of $(PhE)_2PbI_4$ at 10 K is a clear indication of the invalidity of the rigid body approximation in $(PhE)_2PbI_4$ at cryogenic temperatures. The observed mode splitting is a result of the lower symmetry of $(PhE)_2PbI_4$. Due to the lower symmetry, there are twice as many unique Pb and I atoms (which are not related by symmetry) in the unit cell of $(PhE)_2PbI_4$. Lifting the rigid body approximation (*i.e.* including all the degrees of freedom of the organic molecules in the unit-cell) may lift the degeneracy of modes related to different unique atoms, and result in the observed increase in Raman active modes. As temperature increases, the energy difference between modes decreases as well and they appear as a single peak. An alternative explanation for the mode splitting at cryogenic temperatures is a possible smooth phase transition that was never reported, to the best of our knowledge.

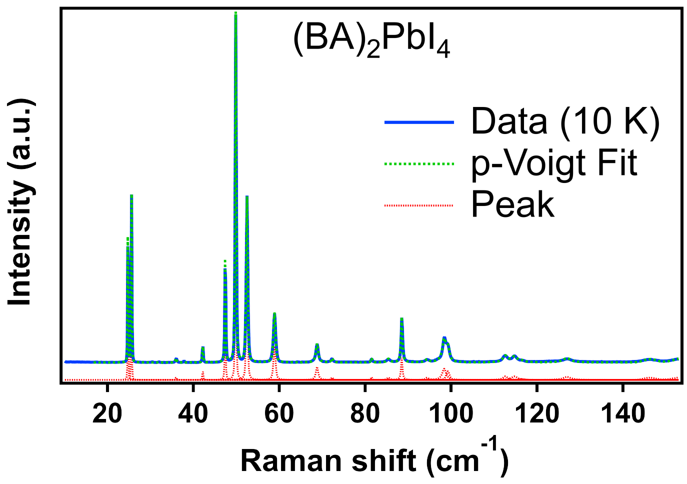

Figure S4. Deconvolution to the multi-pseudo-Voigt model of the unpolarized (summing over all polarizations) Raman spectrum of $(BA)_2PbI_4$ at 10 K. The data (blue trace) and fit result (green trace) are offset from the individual p-Voigt peaks (red traces) for clarity.

S4. Multi-Pseudo-Voigt Deconvolution

Each Raman spectrum was deconvolved to a product of the Bose-Einstein distribution (n_{BE}) and a multi-pseudo-Voigt line shape:

$$I_{Raman}(\omega) = c_{BE} * \sum_i c_i \left[(1-\zeta_i) * \frac{\omega|\omega_{0,i}|\Gamma_i^2}{\omega^2\Gamma_i^2 + (\omega^2 - \omega_{0,i}^2)^2} + \zeta_i * \exp \frac{-(\omega - \omega_{0,i})^2 \ln 16}{\Gamma_i^2} \right] \quad \text{(S1)}$$

$$c_{BE} = n_{BE} + 1 = (e^{\frac{\hbar\omega}{k_b T}} - 1)^{-1} + 1 \quad \text{(S2)}$$

Where $\omega_{0,i}$, c_i and Γ_i are the position, intensity and full-width at half-maximum (FWHM) of each peak respectively, ω is the measured frequency (Raman shift), T is the temperature, $\hbar$ is the Planck constant, k_b is the Boltzmann constant and ζ is the fraction of Gaussian in the pseudo-Voigt peak. The Lorentz oscillator and Gaussian in eq S1 are normalized variation of the Lorentz oscillator and Gaussian distribution where c_i is the intensity of the peak and Γ is the FWHM. In order to reduce the number of fitting parameters, we set ω_i and Γ_i to be the same for both the Lorentz oscillator and the Gaussian, even though the source of the Gaussian may be related to our system's response. Figure S4 shows the deconvolution of the unpolarized spectra of $(BA)_2PbI_4$ at 10 K to 22 oscillators.

The Lorentz oscillator is the conventional model for fitting Raman spectra, since the

scattering signal originates from the imaginary part of the susceptibility and Γ_i is inversely related to the life-time of the mode. The width of peaks at 10 K approached our system resolution, resulting in inhomogeneous broadening of the peaks, which could be captured only by including the Gaussian distribution. For all the data above 10 K, the Lorentz oscillator model ($\zeta_i = 0$) was used.

S5. PO Raman at 10 K

Polarization Orientation (PO) Raman scattering measurements were conducted in a home-built back-scattering system[10,11] using below band-gap CW lasers with 1 mW excitation power. $(BA)_2PbI_4$ and $(PhE)_2PbI_4$ were measured using 1.58 eV pump-doid laser (Toptica Inc., USA) while $MAPbI_3$ was measured using 1.16 eV solid state Nd:YAG laser

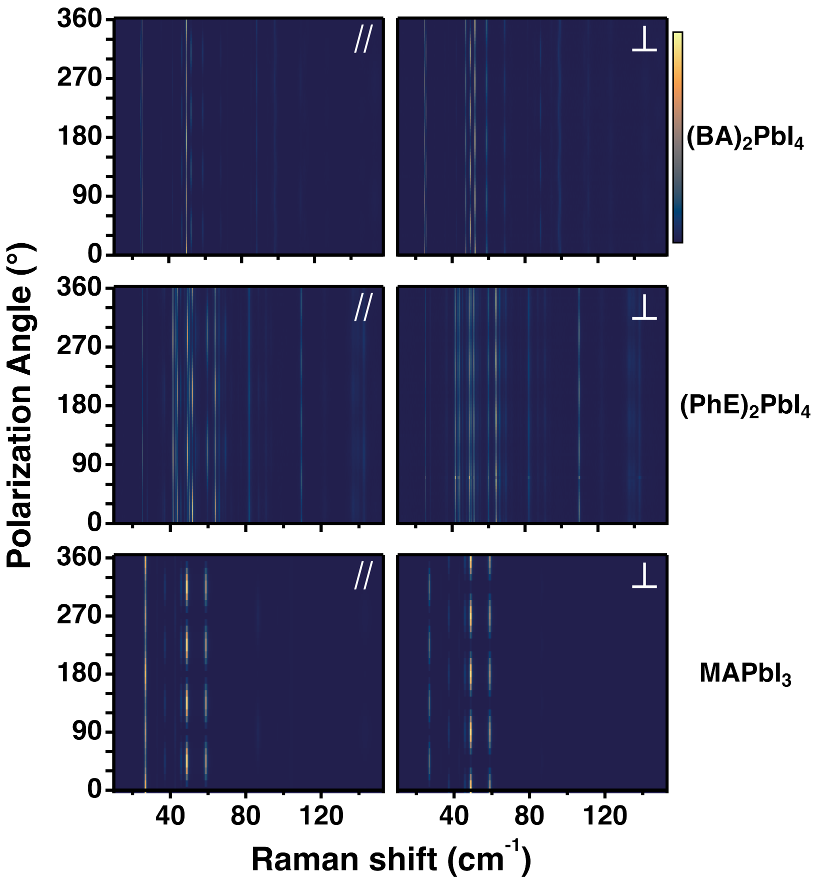

Figure S5. Polarization orientation Raman spectra of $(BA)_2PbI_4$ (top), $(PhE)_2PbI_4$ (middle) and $MAPbI_3$ (bottom) at 10 K, in the parallel (left panels) and perpendicular (right panels) configuration. The color indicates the intensity according to the color-bar on the top right.

(Coherent inc., USA).

The incident beam was linearly polarized by a Glan-laser polarizer (Thorlabs, USA), directed into a microscope (Zeiss, USA) and focused on the sample through 0.55 NA/50x objective (Zeiss, USA). The excitation polarization was controlled by a zero-order half-wave plate (Thorlabs, USA) and was rotated in small increments (10°) between measurements.

The back-scattered beam was collected by the objective and passed through another polarizer to collect only light that was scattered either parallel or perpendicular to the incident polarization. Rayleigh scattering was reduced by passing the beam through volume holographic beam-splitter and two OD>4 notch filters (Ondax Inc., USA).

Finally, the beam was focused to 1 m long spectrometer (FHR 1000, Horiba) dispersed by 1800 gr/mm grating, achieving ≈0.3 cm^{-1} spectral resolution, and detected by Si CCD (Horiba Inc., USA) or liquid-N_2 cooled InGaAs detector (1.16 eV laser).

For temperature control, all crystals were mounted into liquid He cooled optical cryostat (Janis Inc., USA).

Unpolarized spectra were obtained by summing the spectra of all measured incident polarizations collected both in parallel and perpendicular configurations and normalizing to the maximum intensity.

Figure S5 presents contour plots of PO Raman spectra of $(BA)_2PbI_4$ (top), $(PhE)_2PbI_4$ (middle) and $MAPbI_3$ (bottom) at 10 K, in both the parallel (left panels) and perpendicular (right panels) configuration. The intensity of all spectra of the same material were normalized to the highest intensity, of both the parallel and perpendicular configurations. The spectra of $(BA)_2PbI_4$ and $(PhE)_2PbI_4$ in Figure S5 are experimental results. On the other hand, the spectra of $MAPbI_3$ were calculated in a procedure described in section S7.

S6. Fitting PO Dependencies

Raman spectra were deconvolved to individual peaks according to multi-pseudo-Voigt model, as described in section S4, in order to calculate the PO dependence of each mode. Ideally, only the intensity of each peak depends on the incident polarization, while the frequency, FWHM and the Gaussian fraction depends mainly on temperature. Therefore, ω_i, Γ_i and ζ_i are determined first, by fitting several spectra to eq S1, and then all the

spectra are fitted one-by-one with c_i as the only fitting parameter. The PO dependence is evaluated by calculating the integrated intensity of each mode and plotting it against the incident polarization.

We extract the number of Raman active modes and their symmetries by performing Factor group analysis.[12] The shape of the Raman tensor is determined by the symmetry of the mode. Factor group analysis predicts 24 Raman active modes for $(BA)_2PbI_4$, which scatters light polarized in the (001) plane (12 A_g and 12 B_{1g} modes), with the Raman tensors:

$$\boldsymbol{R}_{A_g} = \begin{pmatrix} a & 0 & 0 \\ 0 & b & 0 \\ 0 & 0 & c \end{pmatrix}, \ \boldsymbol{R}_{B_{1g}} = \begin{pmatrix} 0 & d & 0 \\ d & 0 & 0 \\ 0 & 0 & 0 \end{pmatrix} \tag{S3}$$

and 24 Raman active modes for $(PhE)_2PbI_4$ (all A_g) with the Raman tensors:

$$\boldsymbol{R}'_{A_g} = \begin{pmatrix} a & d & e \\ d & b & f \\ e & f & c \end{pmatrix} \tag{S4}$$

We extract the Raman tensor components by performing a global-fit to the PO dependencies of both the parallel and perpendicular configurations, to the modified Placzek equation (eq 1 in the main text). The incident and scattered polarization vectors, for measurements perpendicular to the (001) plane are:

$$\hat{e}_i = \begin{pmatrix} cos(\theta) \\ sin(\theta) \\ 0 \end{pmatrix}, \ \hat{e}^T_{S,\|} = \begin{pmatrix} cos(\theta) \\ sin(\theta) \\ 0 \end{pmatrix}, \ \hat{e}^T_{S,\perp} = \begin{pmatrix} -sin(\theta) \\ cos(\theta) \\ 0 \end{pmatrix}$$

The Jones matrix in eq 1 is introduced to account for birefrigence effects, due to the anisotropy of the crystal.[13,14] It is given by:

$$\boldsymbol{J} = \begin{pmatrix} 1 & 0 & 0 \\ 0 & e^{i|\phi_y|} & 0 \\ 0 & 0 & e^{i|\phi_z|} \end{pmatrix}$$

Where $|\phi_y|$ and $|\phi_z|$ are the relative phases between the a and b or a and c components,

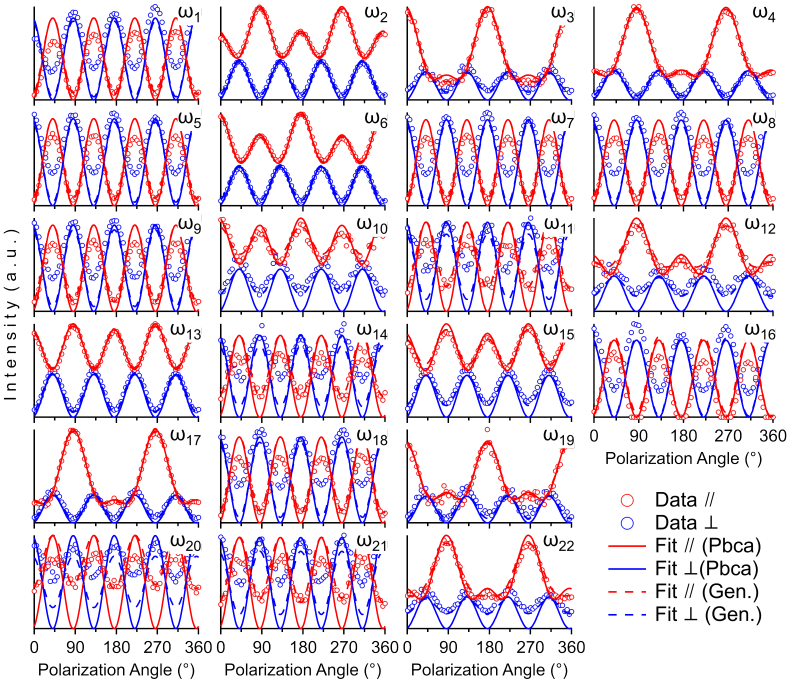

Figure S6. The PO dependencies of the modes of $(BA)_2PbI_4$ at 10 K, in the parallel (red) and perpendicular (blue) configurations. Experimental data represented by circles, the solid and dashed lines are the best global-fit results to eq 1 in the main text, using the tensors in eq S3 and eq S4, respectively.

respectively. Since $(BA)_2PbI_4$ and $(PhE)_2PbI_4$ were measured perpendicular to the (001) plane, we fixed $\left|\phi_z\right| = 0$. Prior to fitting the data, we assigned each mode of $(BA)_2PbI_4$ to a specific symmetry, A_g or B_{1g}, by simulating the PO dependencies according to eq 1 using the Raman tensors above and comparing the the data and simulations.

Figure S6 presents the PO dependencies of the modes of $(BA)_2PbI_4$ at 10 K and their global-fit results to eq 1, when using the Raman tensors predicted from factor group analysis (space group *Pbca*) and a general Raman tensor (same as the tensor used for $(PhE)_2PbI_4$). The mode index in the ω_i notation was taken from the 10 K deconvolution. The frequency corresponding to each mode is presented in sections S9 and S8.

The same process was used to fit the PO dependencies of $(PhE)_2PbI_4$. The PO dependencies of all the modes of $(PhE)_2PbI_4$ are similar to ω_2 and ω_{13} and are therefore not presented.

S7. Rotating the PO Raman of $MAPbI_3$

The PO Raman spectra of $MAPbI_3$ were generated by reversing the process described in Ref. 11 and in sections S4 and S6. Briefly: First, the PO dependence of each mode was calculated by evaluating eq 1 in the main text as function of incident angle, using the reported Raman tensor and Jones matrix and the appropriate incident and scattered light vectors. Then, the intensity of each mode was calculated from the integrated intensity, using the frequency, FWHM and ζ obtained in the initial deconvolution process. Finally, the spectrum at each polarization angle was calculated by plugging into the pseudo-Voigt equation the frequency, FWHM and ζ, which are polarization independent, and the peak intensity corresponding to the specific excitation angle. The spectra were normalized to the highest intensity in both parallel and perpendicular configurations, stacked together and displayed as contour-plot. Unpolarized spectrum was obtained by summing all the spectra and normalizing to the highest intensity.

$(BA)_2PbI_4$ and $(PhE)_2PbI_4$ were measured parallel to the crystallographic c axis (perpendicular to the (001) plane), and the incident angle was defined as the angle from the crystallographic a axis. In both crystals, a and c axes are the shortest and longest crystallographic vectors, respectively (see section S2). Since the crystal orientation of $MAPbI_3$ and $(BA)_2PbI_4$ are different, it is not enough to calculate the PO Raman of $MAPbI_3$ parallel to the c axis, and the crystal orientation should be matched. Therefore, the PO Raman of $MAPbI_3$ was calculated parallel to the longest crystallographic vector b (perpendicular to the (010) plane) and the incident angle was defined as the angle from the shortest crystallographic vector c. The corresponding incident and scattered vectors are:

$$\hat{e}_i = \begin{pmatrix} sin(\theta) \\ 0 \\ cos(\theta) \end{pmatrix}, \; \hat{e}^T_{S,\parallel} = \begin{pmatrix} sin(\theta) \\ 0 \\ cos(\theta) \end{pmatrix}, \; \hat{e}^T_{S,\perp} = \begin{pmatrix} cos(\theta) \\ 0 \\ -sin(\theta) \end{pmatrix}$$

It is noteworthy that the B_{3g} mode of $MAPbI_3$ at 98.7 cm^{-1} is expected to be inactive perpendicular to the (010) plane.

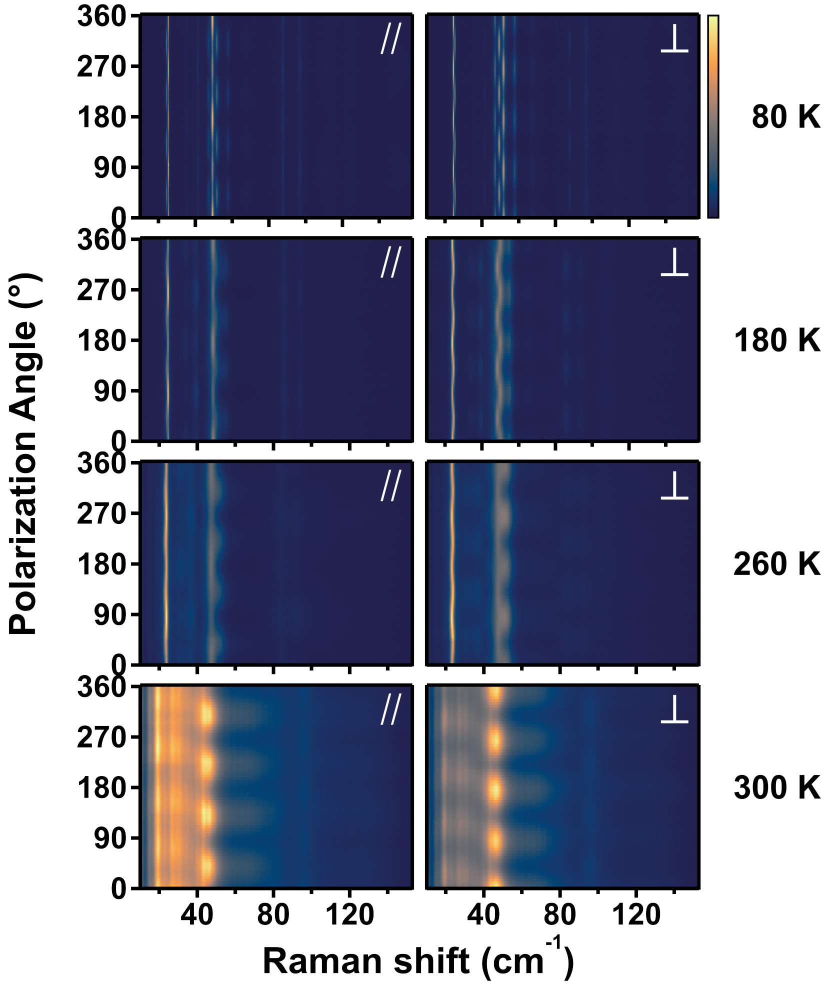

Figure S7. Polarization orientation Raman spectra of $(BA)_2PbI_4$ at (top-to-bottom) 80 K, 180 K, 260 K and 300 K in the parallel (left panels) and perpendicular (right panels) configuration. The color indicates the intensity according to the color-bar on the top right.

S8. Temperature-Dependent PO Raman

In order to properly deconvolve the PO Raman spectra of $(BA)_2PbI_4$ at 300 K we measured temperature dependent and PO Raman spectra of $(BA)_2PbI_4$ below and above the phase transition. Figure S7 presents contour plots of the PO Raman spectra of $(BA)_2PbI_4$ between 80-300 K in the parallel and perpendicular configurations. The fitting process begins by deconvolving the PO Raman at 10 K, followed by deconvolving the temperature dependent Raman spectra while tracking the red-shifting and broadening of the peaks. PO Raman spectra enable us to see modes which otherwise may be hidden.[15] Therefore, at the same time we also deconvolve the PO Raman spectra, making sure we capture the correct number of modes and that their symmetry does not change. We use the frequencies

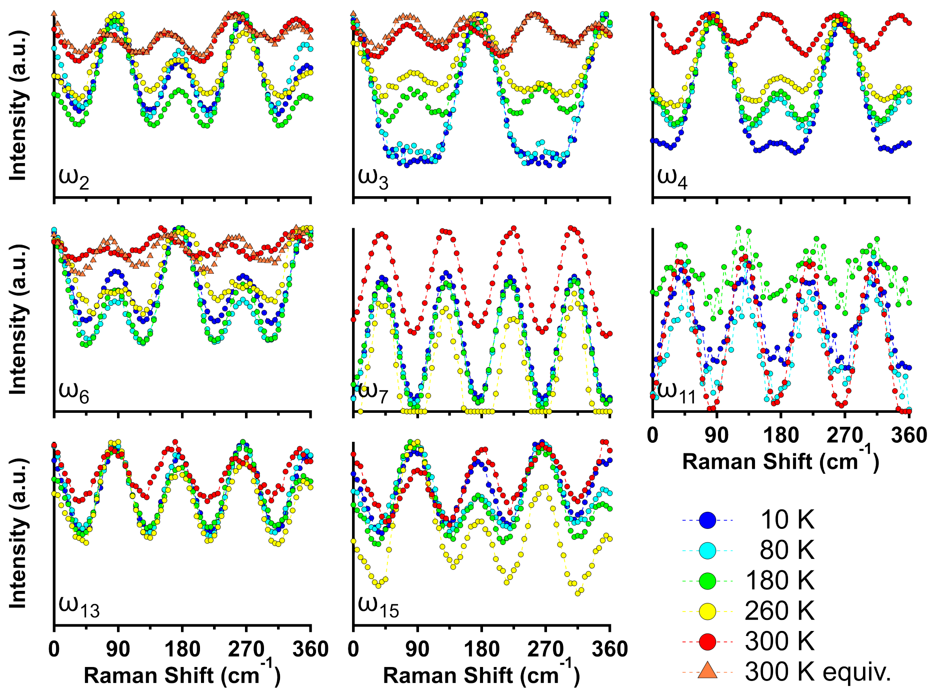

Figure S8. Temperature dependent PO dependencies, in the parallel configuration, of selected modes of $(BA)_2PbI_4$ showing the similar PO dependence above and below the phase transition. Equivalent modes at 300 K (triangles) originate from a different low-temperature phase (see main text). Mode indexes are taken from 10 K spectra. The frequencies are listed in Table S5

at 260 K and the similarities in the spectral shape between 260 K and 300 K, to deconvolve the PO Raman spectra at 300 K. Finally, we use the temperature dependence of each peak and the temperature evolution of the PO dependence to correlate between the peaks below and above the phase transition. Since some of the modes broaden and overlap as temperature increases, we also compared the sum of PO dependencies of overlapping modes to the PO dependencies at 300 K.

Figure S8 presents the temperature evolution of the PO dependencies (parallel configuration) of the modes of $(BA)_2PbI_4$ which were detected in the PO Raman spectra at 300 K, showing that the symmetries and PO dependencies remained the same above and below the phase transition. The corresponding frequencies are presented in Table S5. It is noteworthy that the fit confidence decreases with frequency and temperature, due to peak broadening and decreased intensity. Modes which could not be resolved are presented in blank cells, while low-confidence modes are marked in red. Modes which could not be correlated according to the PO dependencies are marked with superscript # or $*$. The modes at 300 K at 19.6 and 23.0 cm^{-1} (ω_2), 27.1 and 30.7 cm^{-1} (ω_3), and 40.0 and 43.2

Table S5. Frequency of the Raman active modes of $(BA)_2PbI_4$ at various temperature, as extracted PO Raman spectra deconvolution to multi-pseudo-Voigt.

Mode Index	Frequency (cm^{-1}) 10 K	80 K	180 K	260 K	300 K	Symmetry
1	24.8	24.5	23.9	23.1		B_{1g}
2	25.6	25.3	24.7	23.8	19.6<br>23.0	A_g
3	36.0	35.3	33.8	31.5	27.1<br>30.7	A_g
4	42.2	41.5	39.4	36.9	35.4	A_g
5	47.4	47.1	46.2			B_{1g}
6	49.9	49.4	48.4	47.6	40.0 , 43.2	A_g
7	52.5	51.8	49.9	48.4		B_{1g}
8	58.9	57.8	54.8	51.8	46.9	B_{1g}
9	68.8	67.5	66.2	69.0		B_{1g}
10	72.3	72.2		71.4		A_g
11	81.5	80.6	77.0		68.9	B_{1g}
12	85.4	84.3				A_g
13	88.5	87.6	85.7	83.9	82.4	A_g
14	94.4	95.0	89.7			B_{1g}
15	98.4	98.8	94.0	92.5	96.7	A_g
16	99.3					B_{1g}
17	112.6	111.6				A_g
18	114.8	113.9	108.7	106.2		B_{1g}
19	116.4	113.3				A_g
20	127.0	125.0	123.9*	123.9*	129.9	B_{1g}
21	146.3					B_{1g}
22	153.5	149.2	140.5	132.9#		A_g

* Mode assigned according to frequency only. Symmetry does not match.

\# Mode symmetry matches, but the PO dependence does not.

cm^{-1} (ω_6) are suspected as the same motion, originating from the two different wells in the biased double-well potential.

Increased aharmonicity can be shown by calculating the damping ratio of each mode, defined as $\Gamma_i/2\omega_i$. The damping ratio dictates the type of solution when solving the equation of a damped harmonic oscillator: $\Gamma_i/2\omega_i < 1$ result in a damped oscillator while $\Gamma_i/2\omega_i > 1$ is an over-damped oscillator. Figure S9 presents the calculated damping ratio

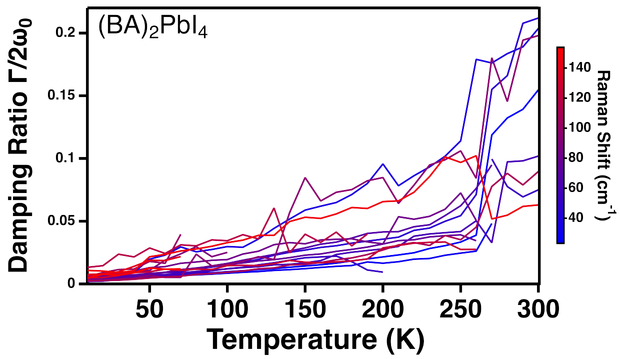

Figure S9. Damping ratio of the Raman active modes of $(BA)_2PbI_4$ as function of temperature, showing the increase in anharmonicity of the low-frequency modes during the phase transition. The color of the trace indicates to the mode's frequency at 10 K, according to the color-scale on the right.

as function of temperature, of the Raman active modes of $(BA)_2PbI_4$. The trace color indicates the mode's frequency at 10 K, redder (bluer) color corresponds to a higher (lower) frequency mode. The classic harmonic oscillator model does not contain damping and it is considered a result of phonon-phonon scattering, a manifestation of anharmonicity.[16] Therefore, the abrupt increase in damping ratio during the phase transition, indicates an increase in phonon-phonon scattering (anharmonicity) in the lower frequency A_g modes.

S9. 2D and 3D Peak Correlation

The correlation process between the modes of $(BA)_2PbI_4$ and $MAPbI_3$ was made in few steps: First we compared the frequencies and symmetries from the spectra at 10 K. We found good agreement of mode symmetries below 80 cm^{-1}, while the comparison above had to rely mostly on frequency and spectral shape. Second, the atomic motions of $(BA)_2PbI_4$ and $MAPbI_3$ were extracted from DFPT analysis. The assignment of the modes of $MAPbI_3$ was already reported by us,[11] and similar comparison was made for the modes of $(BA)_2PbI_4$. Each calculated normal mode of $(BA)_2PbI_4$ corresponds to either A_g or B_{1g} symmetry, as predicted by factor group analysis. Nevertheless, the extracted Raman tensors from DFPT could not be used to relate the calculated and experimental spectra, due to complexity of butyl-ammonium and the low dimensionality. Therefore, only frequencies and symmetries were used to correlate the modes of $(BA)_2PbI_4$. Third, we compared

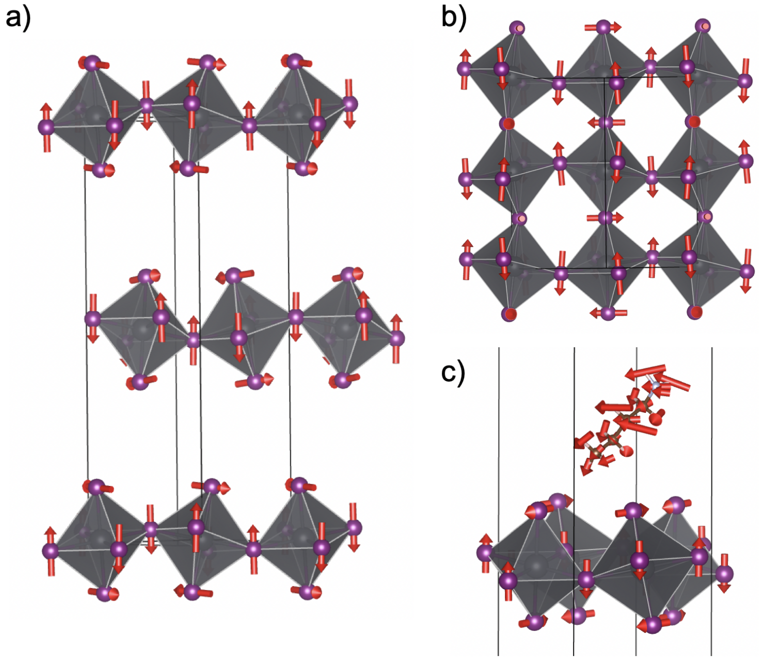

Figure S10. Inorganic atomic motions corresponding to (a) ω_5 of $(BA)_2PbI_4$ and (b) ω_6 of $MAPbI_3$, as calculated by DFPT, showing the similar motions. (c) Zoom in on the motion of an organic cation in ω_5 of $(BA)_2PbI_4$. The motions are presented by arrows, and the size of the arrow is proportional to the motion's amplitude.

the atomic motions of the already correlated modes of $(BA)_2PbI_4$ and $MAPbI_3$ to check the similarity in structural dynamics. We repeated the steps, verified and corrected our analysis to get the best possible correlation. Figures S10a and S10b present the inorganic atomic motion of correlated modes of $(BA)_2PbI_4$ and $MAPbI_3$ respectively. Figure S10c presents the significant contribution on organic molecule to the atomic motion, in the same mode of $(BA)_2PbI_4$ presented in Figure S10a.

Table S6 presents the correlation between experimental and calculated modes of $(BA)_2PbI_4$, the corresponding atomic motions of the inorganic atoms, and the comparison to the modes of $MAPbI_3$ according to both frequency and atomic motions.

The axes mentioned in parentheses are unique axes to describe the motions: the rotation axis; the direction of stretching or scissoring; the direction along which the direction of displacement of I atoms change sign. For possible comparison between similar modes, we used a specific octahedron to describe the motion. The sign of rotational motions was chosen according to the right-hand-rule. One exception is the out-of-plane octahedral tilting of $MAPbI_3$, where adjacent octahedra move in perpendicular directions instead of

opposite directions. Therefore, a notation α_β was chosen, where adjacent octahedra move in the directions $\beta \pm \alpha$. Negative signs are represented with top-bar ($-\alpha \rightarrow \bar{\alpha}$). In order to compare the directions of displacement, the coordinate system of $MAPbI_3$ should be rotated so the longest crystallographic vector (b) would be rotated to the c direction (see section S7). Due to the lower dimensionality of $(BA)_2PbI_4$ compared to $MAPbI_3$, some mode mixing and splitting are observed, as well as modes of $(BA)_2PbI_4$ with no corresponding mode in $MAPbI_3$.

Since the spectrum of $(PhE)_2PbI_4$ at 10 K is complicated, and all the modes have A_g symmetry with a general Raman tensor, we correlated the modes of $(PhE)_2PbI_4$ to those of $(BA)_2PbI_4$ and $MAPbI_3$ according to frequency and spectral shape. We note that above 80 cm^{-1} the modes of $(BA)_2PbI_4$ and $MAPbI_3$ are dominated by motion of the organic cation. In this spectral range, the modes of $(BA)_2PbI_4$, $(PhE)_2PbI_4$ and $MAPbI_3$ have lower intensity and are broader. Moreover, in the region of 80-150 cm^{-1} the difference in the Raman spectra is more significant and the correlation is less confident.

Table S7 presents the comparison between the structural dynamics of $(BA)_2PbI_4$, $MAPbI_3$ and $(PhE)_2PbI_4$ according to temperature-dependent PO Raman. Most modes of $(BA)_2PbI_4$ and $MAPbI_3$ correspond to several modes of $(PhE)_2PbI_4$, due to mode splitting when temperature approaches 10 K.

Table S6: Comparison between experimental (10 K) and calculated (DFPT) modes of $(BA)_2PbI_4$ and the correlation $MAPbI_3$. Only the dominating motions of the inorganic atoms are presented. The atomic motions of $MAPbI_3$ were extracted from DFPT analysis reported in Ref. 11

$(BA)_2PbI_4$				$MAPbI_3$		
$\omega_{Exp.}$ (cm^{-1})	ω_{DFPT} (cm^{-1})	Symmetry	Inorganic Motion	$\omega_{Exp.}$ (cm^{-1})	Symmetry	Inorganic Motion
24.8	22.2	B_{1g}	Tilt ($\bar{b}$)	26.9	A_g	Tilt ($\bar{c}_{\bar{a}}$)<br>Tilt($\bar{a}$) + Twist (b)
25.6	21.8	A_g	Tilt (b) ,<br>Tilt (a)			
36.0	32.6	A_g	Tilt ($\bar{b}$) + Twist (c)	33.0	A_g	Tilt ($c_{\bar{a}}$)<br>Tilt (a)
42.2	39.2	A_g	Tilt ($\bar{b}$)	37.2	B_{2g}	Tilt ($\bar{a}_{\bar{c}}$)
47.4	45.7	B_{1g}	Tilt (a) ,<br>Pb-I_B Wagging (b)	48.8	B_{2g}	Tilt ($a_{\bar{c}}$)<br>Pb-I_B Wagging ($a+b$)
49.9	50.3	A_g	Tilt (a) ,<br>Tilt (b)	42.6	A_g	Tilt ($\bar{c}_{\bar{a}}$) ,<br>Tilt (a) + Twist ($\bar{b}$)
52.5	53.2	B_{1g}	Tilt ($\bar{a}$) ,<br>Tilt ($\bar{a}$)<br>+ Pb-I_B Scissoring (b)	45.8	B_{2g}	Not Predicted by DFPT
58.9	60.7	B_{1g}	Tilt ($a \bar{+} b$) ,<br>Pb-I_B Tilt ($\bar{a}$)<br>+ Pb-I_B Scissoring (b)	58.9	B_{2g}	Tilt ($a_{\bar{c}}$)<br>Pb-I_B Scissoring (a)
68.8	70.4	B_{1g}	Pb-I_T Symmetric Stretch<br>Scissoring (a)			
72.3	71.2	A_g	Pb-I_T Symmetric Stretch	No Correlating Mode		
81.5	73.4	B_{1g}	Pb-I_T Symmetric Stretch			
85.4	78.2	B_{1g}	Pb-I_T Symmetric Stretch			
88.5	88.6	A_g	Pb-I_T Symmetric Stretch<br>Pb-I_B Antisymmetric Stretch ($a+\bar{b}$)	86.5	A_g	Tilt ($c_{\bar{a}}$) ,<br>Twist (b)

94.4	101.3	B_{1g}	Pb-I_T Contract , Pb-I_B Contract	96.7	A_g	Pb-I_B Antisymmetric Stretch $(a+b)$
98.4	99.0	A_g	Tilt $(a+b)$, Pb-I_B Antisymmetric Stretch $(a+b)$	98.7	B_{3g}	Pb-I_T Contract , Pb-I_B Symmetric Stretch
99.3	99.2	B_{1g}	Pb-I_T Contract , Pb-I_B Symmetric Stretch			
112.6	113.0	A_g	Tilt (a) , Tilt (b)	102.7	B_{2g}	Tilt $(\bar{a})$ + Pb-I_B Scissoring (c)
114.8	112.7	B_{1g}	Tilt $(\bar{b})$			
116.4	116.8	A_g	Tilt $(a+b)$, Tilt (c)	104.4	A_g	Tilt $(\bar{a}_c)$, Tilt $(\bar{a})$ + Pb-I_B Scissoring (a)
127.0	136.2	B_{1g}	Tilt $(a+b)$, Tilt $(\bar{a})$ + Scissoring (b)			
146.3	142.2	B_{1g}	Tilt $(a \bar{+} b)$, Pb-I_B Scissoring (a)	No Correlating Mode		
153.5	153.4	A_g	Tilt $(\bar{b})$, Tilt (b) + Twist (c)	143.3	A_g	Tilt $(\bar{c}_a)$, Twist $(\bar{b})$

Table S7. Correlation of the observed Raman active modes of $(BA)_2PbI_4$, $MAPbI_3$ and $(PhE)_2PbI_4$. The frequencies are according to spectral deconvolution at 10 K to multi-pseudo-Voigt model.

$(BA)_2PbI_4$		$MAPbI_3$		$(PhE)_2PbI_4$	
Mode Index	Raman Shift (cm^{-1})	Mode Index	Raman Shift (cm^{-1})	Mode Index	Raman Shift (cm^{-1})
1	24.8	1	26.9	1	20.9
2	25.6			2	23.4
3	36.0	2	33.1	3	31.9
4	42.2	3	37.2	4	32.6
				14	49.3
5	47.4	6	48.8	15	49.9
				16	50.9
				5	37.2
				6	38.6
6	49.9	4	42.6	7	39.6
				8	41.3
				9	43.2
				10	44.1
				11	44.9
7	52.5	5	45.8	12	45.8
				13	47.4
				17	55.4
8	58.9			18	58.2
				19	59.6
		7	58.9	20	61.6
9	68.8			21	63.6
				22	65.0
				21	68.1
				24	71.9
10	72.3			25	73.2
				26	77.6
11	81.5			27	78.8
				28	82.6
12	85.4			29	86.5
				30	89.2
13	88.5	8	86.5	31	105.3
14	94.4	9	96.7		
15	98.4	10	98.7	32	117.7
16	99.3				
17	112.6	11	102.7	33	132.8
18	114.8			34	135.4
19	116.4	12	104.4	35	138.5
20	127.0				
21	146.3				
22	153.3	14	143.3		

References

[1] C. C. Stoumpos, D. H. Cao, D. J. Clark, J. Young, J. M. Rondinelli, J. I. Jang, J. T. Hupp, and M. G. Kanatzidis, Ruddlesden-Popper Hybrid Lead Iodide Perovskite 2D Homologous Semiconductors, Chem. Mater. **28**, 2852 (2016).

[2] K. Wang, C. Wu, D. Yang, Y. Jiang, and S. Priya, Quasi-Two-Dimensional Halide Perovskite Single Crystal Photodetector, ACS Nano **12**, 4919 (2018).

[3] W. Peng, J. Yin, K.-T. Ho, O. Ouellette, M. De Bastiani, B. Murali, O. El Tall, C. Shen, X. Miao, J. Pan, E. Alarousu, J.-H. He, B. S. Ooi, O. F. Mohammed, E. Sargent, and O. M. Bakr, Ultralow Self-Doping in Two-Dimensional Hybrid Perovskite Single Crystals, Nano Lett. **17**, 4759 (2017).

[4] X. Chen, H. Lu, Z. Li, Y. Zhai, P. F. Ndione, J. J. Berry, K. Zhu, Y. Yang, and M. C. Beard, Impact of Layer Thickness on the Charge Carrier and Spin Coherence Lifetime in Two-Dimensional Layered Perovskite Single Crystals, ACS Energy Lett. **3**, 2273 (2018).

[5] D. G. Billing and A. Lemmerer, Synthesis, Characterization and Phase Transitions in the Inorganic–Organic Layered Perovskite-Type Hybrids [$(C_nH_{2n+1}NH_3)_2PbI_4$], n = 4, 5 and 6, Acta Crystallogr., Sect. B: Struct. Sci., Cryst. Eng. Mater. **63**, 735 (2007).

[6] K. Z. Du, Q. Tu, X. Zhang, Q. Han, J. Liu, S. Zauscher, and D. B. Mitzi, Two-Dimensional Lead(II) Halide-Based Hybrid Perovskites Templated by Acene Alkylamines: Crystal Structures, Optical Properties, and Piezoelectricity, Inorg. Chem. **56**, 9291 (2017).

[7] E. Amerling, S. Baniya, E. Lafalce, C. Zhang, Z. V. Vardeny, and L. Whittaker-Brooks, Electroabsorption Spectroscopy Studies of $(C_4H_9NH_3)_2PbI_4$ Organic-Inorganic Hybrid Perovskite Multiple Quantum Wells, J. Phys. Chem. Lett. **8**, 4557 (2017).

[8] T. Dammak, M. Koubaa, K. Boukheddaden, H. Bougzhala, A. Mlayah, and Y. Abid, Two-Dimensional Excitons and Photoluminescence Properties of the Organic/Inorganic (4-$FC_6H_4C_2H_4NH_3)_2[PbI_4]$ Nanomaterial, J. Phys. Chem. C **113**, 19305 (2009).

[9] O. Yaffe, A. Chernikov, Z. M. Norman, Y. Zhong, A. Velauthapillai, A. M. Van Der Zande, J. S. Owen, and T. F. Heinz, Excitons in Ultrathin Organic-Inorganic Perovskite Crystals, Phys. Rev. B: Condens. Matter Mater. Phys. **92**, 045414 (2015).

[10] M. Asher, D. Angerer, R. Korobko, Y. Diskin-Posner, D. A. Egger, and O. Yaffe, Anharmonic Lattice Vibrations in Small-Molecule Organic Semiconductors, Adv. Mater. **32**, 1908028 (2020).

[11] R. Sharma, M. Menahem, Z. Dai, L. Gao, R. Korobko, I. Pinkas, A. M. Rappe, O. Yaffe, T. M. Brenner, L. Yadgarov, J. Zhang, Y. Rakita, R. Korobko, I. Pinkas, A. M. Rappe, and O. Yaffe, Lattice Mode Symmetry Analysis of the Orthorhombic Phase of Methylammonium Lead Iodide Using Polarized Raman, Phys. Rev. Mater. **4**, 051601 (2020).

[12] D. L. Rousseau, Bauman R. P., and S. P. S. Porto, Normal Mode Determination in Crystals, J. Raman Spectrosc. **10**, 253 (1981).

[13] C. Kranert, C. Sturm, R. Schmidt-Grund, and M. Grundmann, Raman Tensor Elements of β-Ga_2O_3, Scientific Reports **6**, 10.1038/srep35964 (2016).

[14] C. Kranert, C. Sturm, R. Schmidt-Grund, and M. Grundmann, Raman Tensor Formalism for Optically Anisotropic Crystals, Phys. Rev. Lett. **116**, 127401 (2016).

[15] R. Sharma, Z. Dai, L. Gao, T. M. Brenner, L. Yadgarov, J. Zhang, Y. Rakita, R. Korobko, A. M. Rappe, and O. Yaffe, Elucidating the Atomistic Origin of Anharmonicity in Tetragonal $CH_3NH_3PbI_3$ with Raman Scattering, Phys. Rev. Mater. **4**, 092401 (2020).

[16] N. W. Ashcroft and N. D. Mermin, Anharmonic Effects in Crystals, in *Solid State Physics* (Sounders College, New York, 1976) pp. 489–508, College ed.
